# Photodynamic inactivation of *Staphylococcus aureus* with rose bengal reduces superantigen activity

**DOI:** 10.3389/fimmu.2025.1655244

**Published:** 2025-10-02

**Authors:** Patrycja Ogonowska, Adrian Kobiela, Anna Hulacka, Danuta Gutowska-Owsiak, Joanna Nakonieczna

**Affiliations:** ^1^ Laboratory of Photobiology and Molecular Diagnostics, University of Gdańsk and Medical University of Gdańsk, Intercollegiate Faculty of Biotechnology, Gdańsk, Poland; ^2^ Laboratory of Experimental and Translational Immunology, University of Gdańsk and Medical University of Gdańsk, Intercollegiate Faculty of Biotechnology, Gdańsk, Poland; ^3^ Department of Pathomorphology, The University Hospital, Kraków, Poland

**Keywords:** bacterial enterotoxins, MRSA, antimicrobial photodynamic therapy, bacterial colonization, atopic dermatitis, rose bengal

## Abstract

Atopic dermatitis (AD) is a chronic inflammatory skin disorder marked by barrier dysfunction and immune dysregulation. Colonization of lesional skin by *Staphylococcus aureus*, present in up to 80–100% of cases, exacerbates inflammation, in part through production of superantigenic toxins. While standard treatments such as topical corticosteroids, antibiotics, and antiseptic baths are widely used, their outcomes remain variable and often inadequate, highlighting the need for alternative strategies that minimize adverse effects and resistance development. In this study, we evaluated antimicrobial photodynamic inactivation (aPDI) using rose bengal (RB), a photosensitizer activated by visible light, as a potential approach to reduce *S. aureus* colonization and virulence. Across *in vitro*, *ex vivo*, and murine *in vivo* models, RB-mediated aPDI significantly decreased *S. aureus* viability and markedly attenuated the expression and activity of staphylococcal enterotoxins. Transcript and protein analyses confirmed substantial reductions in superantigenic activity post-aPDI. These effects were dependent on the combination of both RB and light, with no significant impact observed with either component alone. Our findings indicate that RB-based aPDI may represent a promising non-antibiotic approach to limit *S. aureus* viability and toxin activity in the context of AD. Our data contribute to the understanding of how photodynamic inactivation affects *S. aureus* virulence and highlight a model for studying the impact of microbial factors on skin immune responses in AD.

## 1 Introduction

*Staphylococcus aureus* is a versatile opportunistic pathogen associated with a wide spectrum of skin and soft tissue infections (SSTIs), including abscesses, furuncles, impetigo, and staphylococcal scalded skin syndrome (SSSS). In addition to these superficial infections, *S. aureus* is capable of causing severe invasive diseases such as osteomyelitis, infective endocarditis, septic arthritis, and pneumonia. In many cases, the skin is the primary site where infection begins, typically after the epidermal barrier is breached, allowing bacteria to invade deeper tissues (1). The predominance of *S. aureus* in skin infections is largely attributed to its diverse arsenal of virulence factors with immunomodulatory properties (2). Pathogenesis involves multiple strategies, including disruption of epithelial integrity, inactivation of antimicrobial peptides (AMPs) - such as dermicidin, human β-defensins (hBDs), and cathelicidin (LL-37) - enhanced adhesion to keratinocytes, and cytotoxicity toward neutrophils (3, 4). These innate immune evasion mechanisms collectively facilitate persistent colonization. In addition, *S. aureus* employs strategies to circumvent adaptive immunity, particularly through interference with T-cell responses (5–8).

Among its numerous virulence determinants, *S. aureus* secretes exoenzymes (lipases, nucleases, proteases) and a wide range of exotoxins, which collectively degrade host tissues and liberate nutrients that sustain bacterial growth. These exotoxins include cytolysins, exfoliative toxins (ETA, ETB), and a group of potent immunomodulators known as superantigens (SAgs) (9, 10). SAgs encompass classic enterotoxins, including staphylococcal enterotoxin A (SEA), staphylococcal enterotoxin B (SEB), staphylococcal enterotoxin C (SEC), staphylococcal enterotoxin D (SED), staphylococcal enterotoxin E (SEE), and staphylococcal enterotoxin I (SEI); enterotoxin-like proteins (SEl-G, through SEl-U); and toxic shock syndrome toxin-1 (TSST-1) (11). Unlike conventional antigens, SAgs activate T-cells by bridging major histocompatibility complex class II (MHC II) molecules and T-cell receptors (TCRs) outside the antigen-specific site, leading to massive polyclonal T-cell activation (12). This interaction results in excessive T-cell proliferation and vast cytokine release from CD4+ T-cells, including IL-2, TNF-β, and IFN-γ, as well as IL-1β and TNF-α from macrophages (13, 14). Clinically, SAgs have been implicated in T-cell resistance to corticosteroids in atopic dermatitis, posing therapeutic challenges (15). Furthermore, their role in exacerbating inflammatory skin conditions complicates disease management (16).

Methicillin-resistant *S. aureus* (MRSA) is a major cause of recurrent SSTIs and is resistant to most β-lactam antibiotics, including cephalosporins, carbapenems, monobactams (17, 18). MRSA colonization is more prevalent in individuals with atopic dermatitis compared to healthy carriers (19, 20). Notably, MRSA isolates from atopic dermatitis produce higher levels of SAgs than the methicillin-sensitive strains (MSSA) (13).The increasing incidence of antibiotic resistance in *S. aureus* underscores the urgent need for alternative therapeutic strategies. One such approach is antimicrobial photodynamic inactivation (aPDI), which combines a non-toxic photosensitizer (PS), specific wavelength light and molecular oxygen to generate reactive oxygen species (ROS) (21). These ROS, including hydroxyl radicals (•OH), superoxide anions (•O_2_^-^) and singlet oxygen (¹O_2_), damage proteins, lipids, nucleic acids, and the bacterial cell membranes, ultimately resulting in bacterial death. Importantly, aPDI does not induce conventional resistance; although bacterial tolerance has been reported (22). *In vitro* and *in vivo* studies validated the efficacy of aPDI against *S. aureus*, prompting the exploration of novel photosensitizer derivatives to enhance therapeutic outcomes (23).

In addition to direct bactericidal effect, aPDI inactivates bacterial virulence factors (24). For example, photodynamic treatment with methylene blue (MB) and red light (λ_max_ = 665 nm) has been shown to inhibit *S. aureus* V8 protease, α-hemolysin, and sphingomyelinase activity (25). Likewise, enterotoxigenic *S. aureus* strains virulence factors have been effectively photoinactivated using the Tetra-Py+-Me in combination with white light (380–700 nm) (26).

Two PSs of particular interest are rose bengal (RB) and new methylene blue (NMB), which predominantly induce type II photochemical reaction, converting molecular oxygen (^3^O_2_) into cytotoxic ^1^O_2_ (27, 28). RB exhibits high biocompatibility, a key criterion for therapeutic application (29). It is routinely used in brucellosis diagnostics (30) and ophthalmology for detecting corneal epithelial damage (31).

RB is activated by green light, which penetrates only the superficial skin layers, making it well suited for cutaneous applications with minimal discomfort (32). RB-mediated aPDI has demonstrated efficacy against both planktonic and biofilm forms of Gram-negative and Gram-positive bacteria, including *S. aureus, Enterococcus hirae*, *Listeria innocua*, and *Streptococcus agalactiae*, and *Escherichia coli* (33–35). NMB, a phenothiazinium dye activated by red light, offers deeper tissue penetration, making it suitable for subcutaneous infections (36). Photodynamic treatment with NMB has been effective in reducing *Candida albicans* infections in skin abrasive wounds and significantly lowered the burden of multidrug-resistant *Acinetobacter baumannii* in both *in vitro* (>6 log_10_ CFU reduction), and *in vivo* (mouse burn model, >3 log_10_ CFU reduction) (37, 38). Additionally, Misba et al. demonstrated that NMB-mediated aPDI is effective against both Gram-positive (*Enterococcus faecalis*) and Gram-negative (*Klebsiella pneumoniae*) bacteria in planktonic and biofilm cultures (39). However, the impact of aPDI on *S. aureus* superantigens remains poorly characterized.

In this study, we investigated the effect of aPDI using RB (green light) and NMB (red light) on five clinically relevant *S. aureus* superantigens - SEA, SEB, SEC, SED, and TSST-1. We assessed both their expression and biological activity post-treatment. Furthermore, we evaluated the efficacy of aPDI and its modulatory effect on virulence factors activity in both an *ex vivo* porcine skin model and an *in vivo* murine model of *S. aureus* skin colonization.

## 2 Materials and methods

### 2.1 Bacterial strains and growth conditions

*Staphylococcus aureus* reference strains were generously provided by Dr Joanna Empel from the National Medicines Institute (NMI), Warsaw, Poland. These strains were analyzed for toxin genes and genetic background (**Table 1**). *S. aureus* was cultured in 5 mL tryptic soy broth (TSB, bioMérieux, France) under aerobic conditions at 37°C with shaking (150 rpm, Innova 40, New Brunswick Scientific, Sweden) for 16–20 h.

**Table 1:** Genetic characterization of the *S. aureus s*trains used in the study.

NMI collection number	Phenotype	*spa* type	ST	CC	*agr*	Toxin genes
10798/11	MSSA	t127	ST1	CC1	3	*sea, seh, selk, selq*
140/05	MSSA	t529	ST59	CC59	1	*seb, selk, selq*
1947/05	MSSA	t015	ST45	CC45	1	*sec, seg, sei*
1005/05	MSSA	t008	ST8	CC8	1	*sed, tst*
Xen40	MSSA	t012	ST30	CC30	3	*sea, tst*

MSSA, methicillin sensitive Staphylococcus aureus; spa, Staphylococcus aureus protein A; ST, sequence type; CC, clonal complex; agr, accessory gene regulator

### 2.2 Eukaryotic cell lines

The eukaryotic cell lines used in the study were (1): shFLG HaCaT, cells with reduced expression of the filaggrin gene (a line transduced with lentiviral particles containing short RNA with a “hairpin” structure (sc-43364-V, Santa Cruz) and (2), shC HaCaT, cell line with an empty vector introduced by transfection, served as control (sc-108080, Santa Cruz) (40).

### 2.3 Antimicrobial photodynamic inactivation

#### 2.3.1 Light source

Illumination was performed using three custom-built LED-based lamps emitting (1) green light (λ_max_ = 515 nm, 35 mW/cm^2^ irradiance; *in vitro*) (2), green light (λ_max_ = 530–535 nm, 10.6 mW/cm^2^ irradiance; *ex vivo* and *in vivo*) and (3) red light (λ_max_ = 632 nm, 20 mW/cm^2^ irradiance) (EMD Technology, Warsaw, Poland; Cezos LED modules, Gdynia, Poland). Light source characteristics were previously published (41).

#### 2.3.2 Chemicals

Rose bengal (RB, 4,5,6,7-tetrachloro-2′,4′,5′,7′-tetraiodofluorescein disodium salt) and new methylene blue (NMB, 3,7-bis(ethylamino)-2,8-dimethylphenothiazin-5-ium chloride) (Sigma-Aldrich (Germany) were dissolved in sterile Milli-Q water, and stored in the dark at -20°C. Before use, stock solutions were thawed and diluted in sterile Milli-Q water; working solutions were stored in the dark at 4°C for up to one month.

#### 2.3.3 Sublethal aPDI treatment for gene expression analysis

The method was previously described (42). Overnight *S. aureus* cultures were diluted 1:100 in fresh TSB and grown to OD_600_ = 0.5. Cultures (510 µL) were added to 24-well plates under four conditions (1): control (dark) (2), light only (3), photosensitizer only, and (4) aPDI (photosensitizer + light). Photosensitizers (RB: 0.2 - 0.5 µM; NMB: 5 µM) were incubated at 37°C for 10–15 min, then exposed to green (2–10 J/cm²) or red light (17.5–30 J/cm²). RNA samples were collected at 20 and 40 min post-irradiation, mixed with RNAlater, and stored at 37°C for no longer than 24 hours before isolation. Serial dilutions (10^-^¹ - 10^-5^) were plated on TSA for colony counts after 24 h. Experiments were done in triplicate.

#### 2.3.4 RNA isolation and reverse transcription.

Total RNA was extracted using the Blood/Cell RNA Mini Kit (Syngen, Poland) with minor modifications. Bacterial pellets (from 500 µL cultures suspended in 1 mL of the RNAlater, Invitrogen, USA) after centrifugation, were lysed in a buffer (120 mM Tris-HCl (pH 8.0), 2 mM EDTA, 1.2% Triton X-100) containing lysostaphin (2U, 5µL, A&A Biotechnology, Poland). The mixture was vortexed at maximum speed for 20 seconds and incubated in a thermoblock (JWE Electronic, Poland) at 37°C for 30 minutes, with brief vortexing (15–20 seconds) every 10 minutes. Subsequent steps were carried out according to the manufacturer’s protocol. On-column DNase digestion was performed using RNase-Free DNase Set (Qiagen, Germany). RNA was eluted using 50 µL of RNase-free water. The isolated RNA was aliquoted into RNase-free Eppendorf tubes and stored at -80°C until further use.

RNA quality was checked on a 1.5% agarose gel and quantified with a NanoDrop 1000 (ThermoFisher Scientific, USA). cDNA was synthesized using the TranScriba Kit (A&A Biotechnology, Poland). In an RNase-free sterile tube, 100 ng of total bacterial RNA was mixed with 1 µL of random hexamer primers and RNase-free water to a final volume of 9.5 µL. The mixture was briefly centrifuged and incubated at 65°C for 5 minutes to denature the RNA template. Subsequently, the following reagents were added: 4 µL 5× reaction buffer, 0.5 µL RNase inhibitor, 2 µL dNTP mix, and 4 µL TranScriba enzyme. Reverse transcription was performed in a GeneAmp PCR System 9600 (Perkin-Elmer, USA) under the following conditions: 25°C for 5 min (primer annealing), 42°C for 60 min (extension), 70°C for 5 min (termination), then held at 4°C. The resulting cDNA was stored at -20°C until further analysis.

#### 2.3.5 qPCR

Toxin gene expression was quantified via qPCR (LightCycler^®^ 480 II) using specific primers (**Table 2**) and Fast SG qPCR Master Mix (EURx, Poland). Expression was normalized to stable reference genes (*gmk*, *ftsZ* for RB/green light; *fabD*, *proC* for NMB/red light) and calculated using the Pfaffl method (43), reported in log_2_ units. All reactions were run in triplicate, and qPCR efficiency was validated (**Supplementary Table S3**).

**Table 2:** Reference genes and target genes used in the study.

Gene	Sequences of primers (5’-3’)	Amplicon length (bp)	References
*fabD*	F: CCT TTA GCA GTA TCT GGA CC R: GAA ACT TAG CAT CAC GCC	102	(44)
*ftsZ*	F: TAT TAC TGG TGG CGA GTC A R: AGT ATT TAC GCT TGT TCG GA	223	(45)
*gmk*	F: AAT CGT TTT ATC AGG ACC R: CTT CAC CTT CAC GCA TTT	120	(46)
*proC*	F: GGC AGG TAT TCC GAT TG R: CTT CCG GTG ATA GCT GTT A	231	(45)
*sea*	F: AAA ATA CAG TAC CTT TGG AAA CGG TT R: TTT CCT GTA AAT AAC GTC TTG CTT GA	92	(47)
*seb*	F: ACA CCC AAC GTT TTA GCA GAG AG R: CCA TCA AAC CAG TGA ATT TAC TCG	81	(47)
*sec*	F: AAT AAA ACG GTT GAT TCT AAA AGT GTG AA R: ATC AAA ATC GGA TTA ACA TTA TCC ATT C	80	(47)
*sed*	F: TGA TTC TTC TGA TGG GTC TAA AGT CTC R: GAA GGT GCT CTG TGG ATA ATG TTT T	115	(47)
*tst*	F: TCA TCA GCT AAC TCA AAT ACA TGG ATT R: TGT GGA TCC GTC ATT CAT TGT T	88	(48)

F, forward primer; R, reverse primer.

### 2.4 Western blot analysis of protein expression

#### 2.4.1 Sublethal aPDI treatment and protein lysate preparation

*S. aureus* cultures (1:100 in TSB, grown to OD_600_ = 1.7–1.9) were treated under four conditions (1): untreated (2), light only (3), photosensitizer only, and (4) aPDI (photosensitizer + light). RB (0.5 µM) and NMB (200 µM) were added to groups 3 and 4, incubated (37°C, 10–15 min), then irradiated (green: 2–12 J/cm²; red: 27.5 - 32.5 J/cm²). Samples were heated (95°C, 5 min), centrifuged, and supernatants were stored at -20°C, while bacterial cell pellets were discarded.

#### 2.4.2 Total protein concentration measurement

Total protein was measured using the RC DC™ Protein Assay (Bio-Rad) per manufacturer’s instructions. Absorbance was read at 750 nm with a SPECORD 2000 PLUS spectrophotometer.

#### 2.4.3 SDS-PAGE and Western blot

Proteins (10 µg of total supernatant proteins) were separated on 12% SDS-PAGE alongside molecular weight markers and toxin standards. Electrophoresis was performed at 180 V for 60 min. Gels were Coomassie-stained if needed. Proteins were then transferred onto PVDF membranes (100 V, 60 min, on ice). Membranes were blocked for 30 minutes at room temperature in 30 mL TBS-Tween (TBST) containing 1% skim milk with gentle shaking. Following two washes with 30 mL of TBST, membranes were incubated overnight at 4°C with primary antibodies (anti-SEA LAI101, LOT#101314AI; anti-SEB LBI202, LOT #92514BI, anti-SEC LCI111, LOT#101012CI, anti-SED LDI303, LOT#70918DI, anti-TSST-1 LTI101, LOT#72617TI; Toxin Technology, Inc., USA) diluted 1:10,000 in TBST with 1% skim milk. After washing, membranes were incubated with HRP-conjugated AffiPure Alpaca Anti-Rabbit IgG secondary antibody (Jackson ImmunoResearch Laboratories Inc., USA) diluted 1:10,000 in the same buffer. Chemiluminescent signals were developed using Clarity Max ECL (Bio-Rad, USA) and imaged with a ChemiDoc XRS+ system (Bio-Rad, USA).

### 2.5 Proliferation assay

Ethical approval was granted by the Medical University of Gdańsk (NKBBN/621-574/2020). PBMCs were isolated from buffy coats of healthy donors via Lymphoprep gradient centrifugation. Cells from three donors were stained with CellTrace™ Far Red and seeded in 96-well plates (2 × 10^5^ cells/well) in RPMI-1640 with 10% FBS and antibiotics.

A mix of PBS, photosensitizer (RB: 0.5 or 5 µM; NMB: 5 µM), and staphylococcal toxin (3.2 µg/mL) was prepared. Toxins were irradiated with green (515 nm, 10–40 J/cm²) or red light (632 nm, 25 J/cm²). Controls included toxin + light, toxin + photosensitizer (dark), untreated toxin, and heat-inactivated toxin. Treated toxins (5 µL, final 80 ng/mL) were added to PBMCs; unstimulated cells served as negative controls.

After six days, cells were stained with CD3-PE, fixed, and analyzed by flow cytometry (InCyte) to assess T-cell proliferation.

### 2.6 ROS detection

ROS generation was assessed using HPF fluorescence in response to hydroxyl radicals. Toxins (SEA, SEB, SEC, SED, TSST-1; 3.2 µg/mL) were combined with PBS, HPF (5 µM), and photosensitizer (RB or NMB, 5 µM) in black 96-well plates. After dark incubation (RB: 10 min; NMB: 15 min), samples were irradiated (green: 515 nm, 40 J/cm²; red: 632 nm, 32.5 J/cm²). Fluorescence was recorded at 490/515 nm using an EnVision Plate Reader.

### 2.7 MTT assay for photo- and cytotoxicity

shFLG HaCaT and shC HaCaT cells (40) were seeded at 1 × 10^4^ cells/well in two 96-well plates (light and dark conditions) and incubated for 24 h in standard conditions. Cells were treated with RB/NMB (10–15 min, dark), washed and irradiated with:

- green light (530–535 nm, 10.6 mW/cm², 6.36 J/cm², 10 min)
- red light (632 nm, 20 mW/cm², 32.5 J/cm², 45 min)

After 24 h, cell viability was assessed using the MTT assay. Formazan absorbance (550 nm) was measured using a Victor Multilabel Plate Reader (PerkinElmer, USA). Viability (%) was expressed as a ratio of treated to untreated samples. Experiments were performed in triplicate, with four technical replicates per condition.

### 2.8 *Ex vivo* porcine skin colonization model

#### 2.8.1 Bacterial colonization of porcine skin

Porcine skin (2 × 2 cm), prepared following Maisch et al. (49), was cleaned, disinfected, and placed on Hepes agar. Porcine skin samples were inoculated with *S. aureus* Xen40 (10^7^ CFU/mL; Perkin Elmer, USA) and incubated at 37°C for 24 h, resulting in visible discoloration (**Supplementary Figure S5**). Four experimental conditions were tested:

1. L (–) PS (–) – untreated, dark
2. L(+) – light only
3. PS(+) – photosensitizer (PS) only
4. aPDI – PS + light

RB (35 µM, 10 µL) was applied to PS(+) and aPDI groups, followed by 30 min dark incubation. Light-treated groups were irradiated with green light (530–535 nm, 6.36 J/cm², 10 min), then incubated 40 min at 37°C.

#### 2.8.2 RNA, protein, and bacterial viability analysis from porcine skin

Forty minutes post-aPDI, skin samples were swabbed for RNA extraction using RNAlater-moistened swabs. Swabs were incubated in a lysis buffer (20 mM Tris-HCl pH 8.0; 2mM EDTA pH 8.0; 1.2% Triton X-100) with lysostaphin (37°C, 40 min), followed by RNA isolation, cDNA synthesis, and qPCR (per Sections 2.4 & 2.5).

For protein analysis, PBS-moistened swabs were collected at 40 min and 24 h, vortexed in PBS, centrifuged (14,000; 5 min), and supernatants were transferred to sterile Eppendorf tubes and mixed with 2x Laemmli buffer, heated, and stored at -20°C. Pellets were discarded. Protein analysis followed Section 3.2 and 3.3.

Bacterial viability was assessed by plating 10^-^¹–10^-8^ serial dilutions from resuspended pellets on TSA. Colonies were counted after 24 h at 37°C.

### 2.9 *In vivo* mouse model of *Staphylococcus aureus* skin colonization

#### 2.9.1 Animal model and experimental groups

The study used 8-week-old female BALB/c mice (n=36, Charles River Laboratories, Germany), under controlled conditions (22°C ± 2°C, 55% ± 5% humidity, 12-h light/dark cycle). Procedures were approved by the II Local Ethical Committee for Animal Experiments in Kraków, Poland (No. 101/2021, April 8, 2021). Mice were divided into six groups:

1. Tape-stripping only (skin repair, n=6)
2. Tape-stripping + *S. aureus* (n=6)
3. Tape-stripping + *S. aureus* + rose bengal (RB, 50 µM) (n=6)
4. Tape-stripping + *S. aureus* + aPDI (single treatment) (n=6)
5. Tape-stripping + *S. aureus* + aPDI (double treatment) (n=6)
6. Tape-stripping + *S. aureus* + green light (λ_max_=530–535 nm) (n=6)

#### 2.9.2 Tape-stripping procedure and skin colonization

On the first day, a 2 cm² dorsal area was shaved, depilated, and subjected to 10–12 tape applications (Omnifix^®^ Elastic, Hartmann, Germany), fresh tape each time until redness appeared, avoiding bleeding. A bioluminescent *S. aureus* Xen40 strain (10^7^ CFU/mL) was applied (groups 2 - 6) under a Tegaderm™ dressing (3M™, USA) to prevent drying. Colonization was monitored via IVIS bioluminescence imaging (Perkin-Elmer, USA).

#### 2.9.3 *In vivo* experimental design

For Group 1, daily observations and photographic documentation of the tape-stripped skin were performed. Daily imaging (IVIS, Perkin-Elmer, USA) was performed under isoflurane anesthesia (3 - 4% induction, 1.5 - 3% maintenance).

Treatments on day 2, included:

Group 3: RB (10 µl, 50 µM) applied for 30 min. under a foil dressing (Tegaderm™, 3M™, USA).
Groups 4 & 5: aPDI - RB (10 µl, 50 µM) applied for 30 min., followed by 10 min irradiation (Group 5 received a second treatment on day 3).
Group 6: green light (λ_max_=530–535 nm) exposure for 10 min.

On day 5, skin swabs (groups 2, 5, and 6) were collected for bacterial protein analysis following the *ex vivo* porcine skin colonization model protocol (section 7.2). Mice were euthanized, and samples were formalin-fixed, H&E stained, and examined (Olympus OlyVIA ver.3.3, Olympus Soft Imaging Solutions GmbH) for inflammation, neutrophil infiltration, and bacterial presence.

#### 2.9.4 Bioluminescence imaging

Bioluminescence was imaged using the IVIS^®^ Lumina III photon-counting system (PerkinElmer, Waltham, MA). Mice were anesthetized with an isoflurane/oxygen mixture and placed on an adjustable stage. Images were acquired in photon-counting mode with an exposure time of 30 s. Bioluminescence signals were quantified within defined regions of interest (ROIs) using the IVIS software and expressed in absolute units (photons·s^-^¹·cm^-^²·sr^-^¹).

### 2.10 Statistical analysis

Statistical analyzes were performed using GraphPad Prism 8 (GraphPad Software, Inc., USA, 2019). One-way analysis of variance (AVOVA) followed by Dunnett’s test for multiple comparisons was used. For all statistical tests, a *p*-value of < 0.05 was considered statistically significant.

## 3 Results

### 3.1 Sublethal aPDI changes the expression of staphylococcal toxin genes

The impact of antimicrobial photodynamic inactivation (aPDI) on the expression of toxin genes was assessed in four *S. aureus* reference strains, each carrying genes for common staphylococcal toxins: 10798/11 (*sea*), 140/05 (*seb*), 1947/05 (*sec*), and 1005/05 (*sed* and *tst*). Two aPDI protocols were employed: (i) rose bengal (RB) with 515 nm light and (ii) new methylene blue (NMB) with 632 nm light. The study aimed to determine whether the observed effects depend on the photosensitizer, light wavelength, or photodynamically induced oxidative stress. Bacteria were exposed to sublethal aPDI conditions, corresponding to a reduction in viable counts of approximately 0.5 log_10_ CFU/mL (please see Supplementary data for the details), and gene expression was quantified via qPCR.

**Figure 1:**
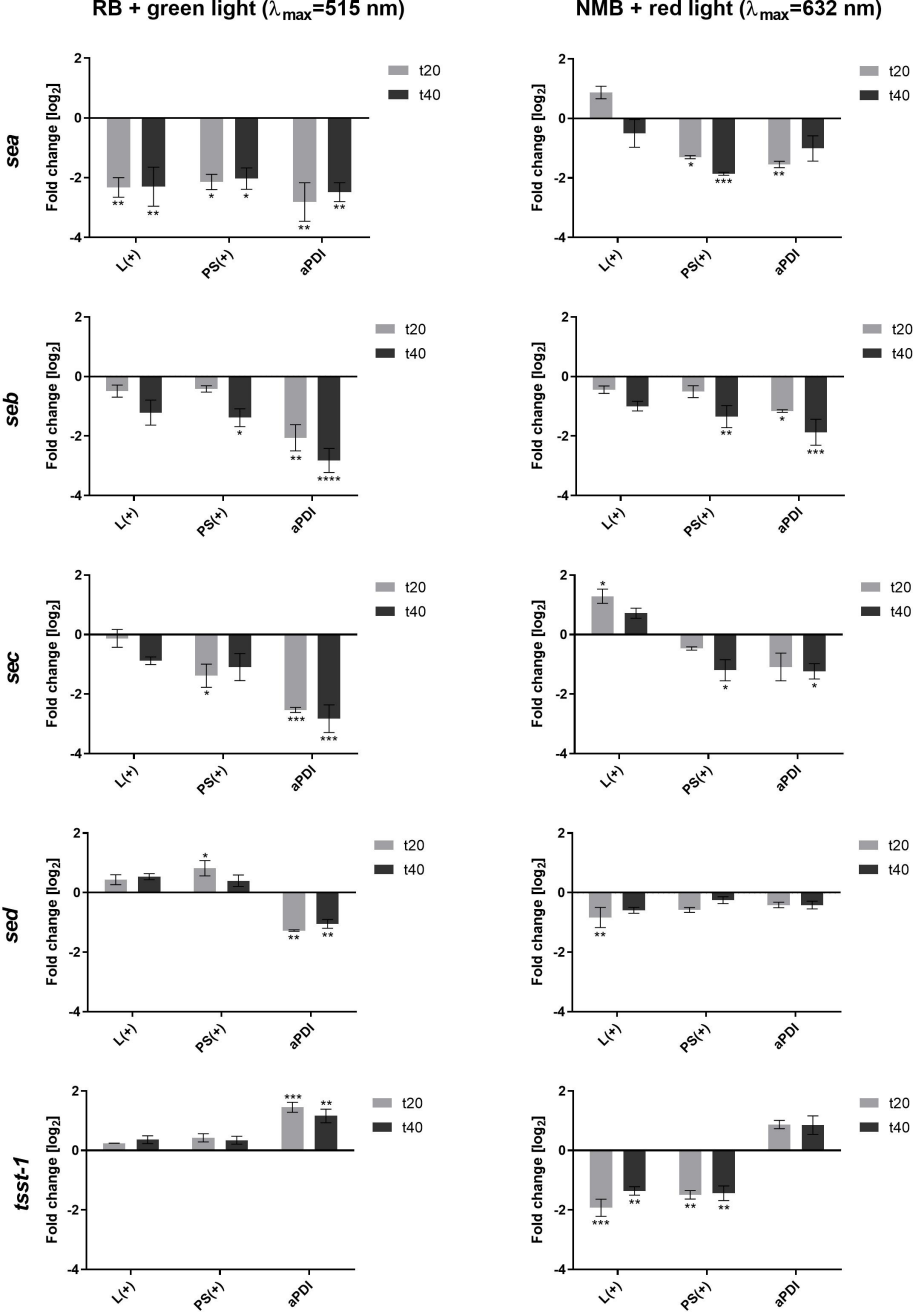
Changes in the expression of staphylococcal toxin genes after sublethal aPDI treatment: relative expression level of *sea*, *seb*, *sec*, *sed* and *tsst*-1 toxin genes after sublethal aPDI treatment. The experiment was performed with either rose bengal (RB) with green light or new methylene blue (NMB) with red light. Significance at respective *p*-values is marked with asterisks [*p < 0.05; **p < 0.01; ***p < 0.001; ****p < 0.0001] with respect to untreated cells (0 J/cm^2^, 0 µM RB/NMB). Error bars represent the standard error of the mean. L(+), bacterial cell treated with light alone (green λ_max_=515 nm/red λ_max_=632 nm); PS(+), cells treated with RB or NMB alone in the dark; aPDI, bacterial cells treated with RB and green light or NMB and red light; t20 and t40, the time points after the irradiation process at which samples were collected.

We first determined the sublethal doses of the compound and light combination (**Supplementary Figure S1**, **Supplementary Table S1**). A significant decrease in the expression of *sea*, *seb*, *sec* and *sed* genes was observed as early as 20 minutes after the treatment, persisting for at least 40 minutes, regardless of the aPDI approach (**Figure 1**). For RB + green light, *sea, seb*, and *sec* expression decreased due to both aPDI and the individual impact of either RB or light, with statistical significance observed only for *sea*. In contrast, NMB + red light significantly downregulated *sea, seb*, and *sec* expression, though red light alone produced variable effects, including both increases (*sec*) and decreases (*seb, sed*).

A distinct expression pattern was observed for *tst*. Unlike the other toxin genes, *tst* expression increased following aPDI, independent of the photosensitizer or light source. Notably, neither RB nor green light alone altered the *tst* expression, while both red light and NMB alone significantly reduced its expression (**Figure 1**). This suggests that *tst* upregulation is directly linked to the photodynamic action rather than to the individual treatment components.

The results demonstrate that aPDI significantly influences toxin gene expression, with effects varying by gene and, to a lesser extent, by the aPDI conditions. This suggests that photogenerated oxidative stress differentially affects gene expression. However, the underlying mechanisms driving these gene-specific responses to photooxidative stress under different aPDI conditions require further investigation.

### 3.2 aPDI inactivates staphylococcal toxin superantigenic function

**Figure 2:**
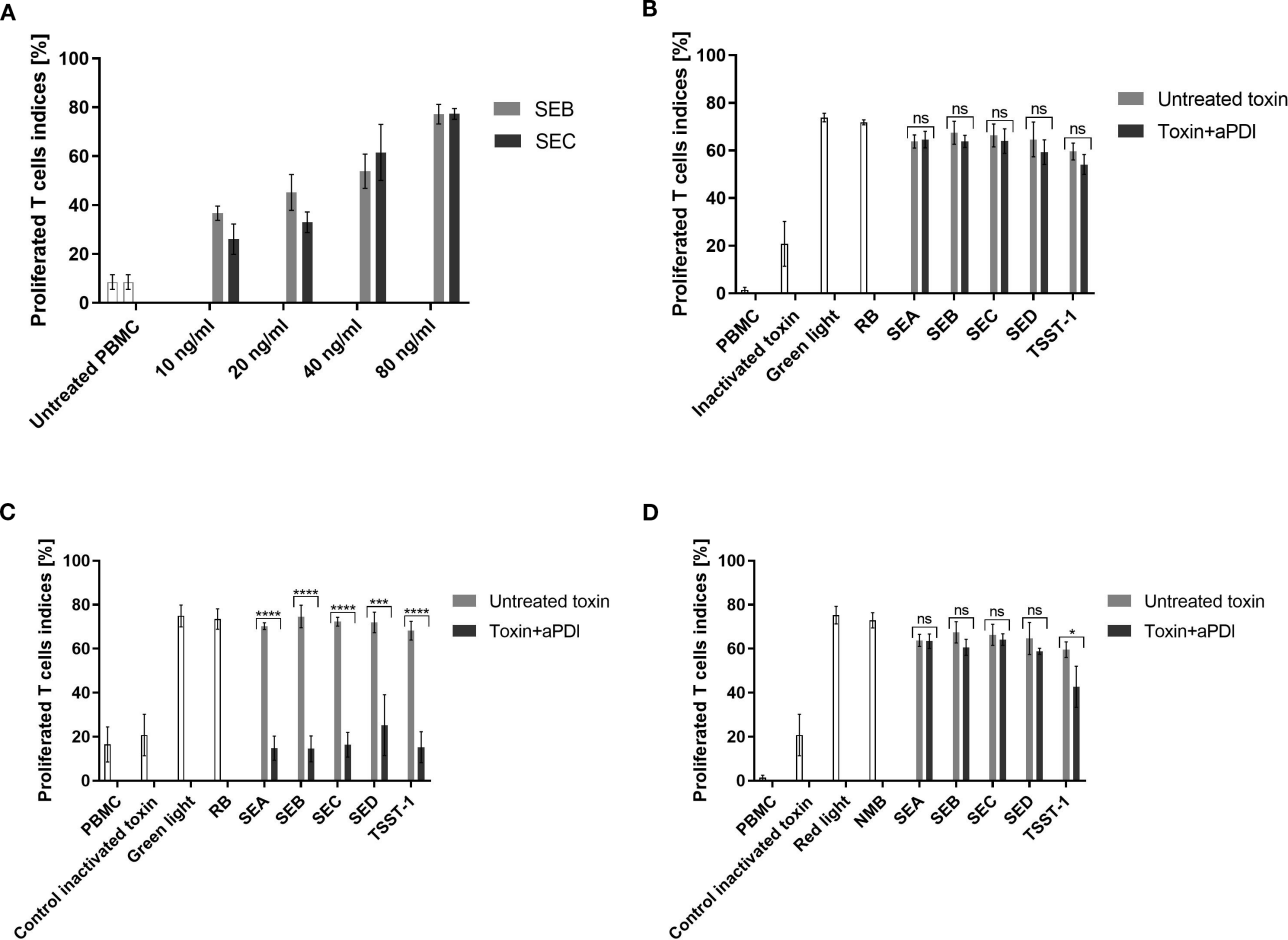
T-cell proliferation assay evaluating toxin activity following aPDI treatment. **(A)** Dose-response curve showing T-cell proliferation induced by staphylococcal toxins. Bars represent mean proliferation indices from three independent donors; error bars indicate standard deviation (SD). Prior to incubation with peripheral blood mononuclear cells (PBMC), toxins were treated as follows: **(B)** sublethal aPDI using rose bengal (RB, 0.5 µM) and green light (515 nm, 35 mW/cm², 12 J/cm²); **(C)** lethal **aPDI u**sing RB (5 µM) and green light (515 nm, 35 mW/cm², 40 J/cm²); **(D)** sublethal aPDI using new methylene blue (NMB, 5 µM) and red light (632 nm, 20 mW/cm², 32.5 J/cm²). Bars represent means ± SD from three donors. Significance at respective *p*-values is marked with asterisks: *p < 0.05; ***p < 0.001; ****p < 0.0001. Toxin controls include heat-inactivated samples (heated at 95°C for 5 min), samples treated with light only (Green light/Red light), samples treated with photosensitizer only (RB/NMB). Toxin designations (SEA, SEB, SEC, SED, TSST-1) refer to proteins treated with antimicrobial photodynamic inactivation (aPDI). ns, non significant.

We then analyzed the effect of aPDI on SEs at the protein level (**Supplementary Figure S2**, **Supplementary Table S2**). Within the limits of our semi-quantitative Western blot analysis, aPDI treatment did not reveal consistent changes in SE protein levels at any time point (**Supplementary Figure S2**). Therefore, we shifted our focus to evaluating its impact on the biological activity of the staphylococcal toxins, particularly their superantigen (SAg) function, which induces T-cell proliferation and cytokine release. The activity of the toxin was assessed using the T-cell proliferation assay. The optimal concentration of the toxin for effective T-cell stimulation was determined to be 80 ng/mL, inducing proliferation in 77% of T-cells (**Figure 2A**). Toxins were treated with: RB and green light or NMB and red light under sublethal (0.5 - 5 µM, 12–32 J/cm²) or lethal (5 µM, 40 J/cm²) conditions. aPDI-treated toxins were then incubated with PBMCs, and T-cell proliferation was measured with light or photosensitizers alone serving as controls.

The impact of aPDI on toxin activity varied depending on the photosensitizer and treatment conditions. RB + green light demonstrated potent toxin inactivation under lethal conditions, reducing T-cell proliferation to near-background levels (SEA: 14.8%, SEB: 14.5%, SEC: 16.4%, SED: 25.3%, TSST-1: 15.2%) (**Figure 2C**). In contrast, sublethal RB treatment had no discernible effect, and neither RB nor green light alone altered toxin activity (**Figure 2B**).

NMB + red light under sublethal conditions, in contrast, was less effective:. Only TSST-1 showed partial reduction (42.7%) while other toxins remained active (**Figure 2D**). Stronger NMB-aPDI conditions were not tested due to phototoxicity toward eukaryotic cells.

### 3.3 aPDI treatment results in ROS generation

**Figure 3:**
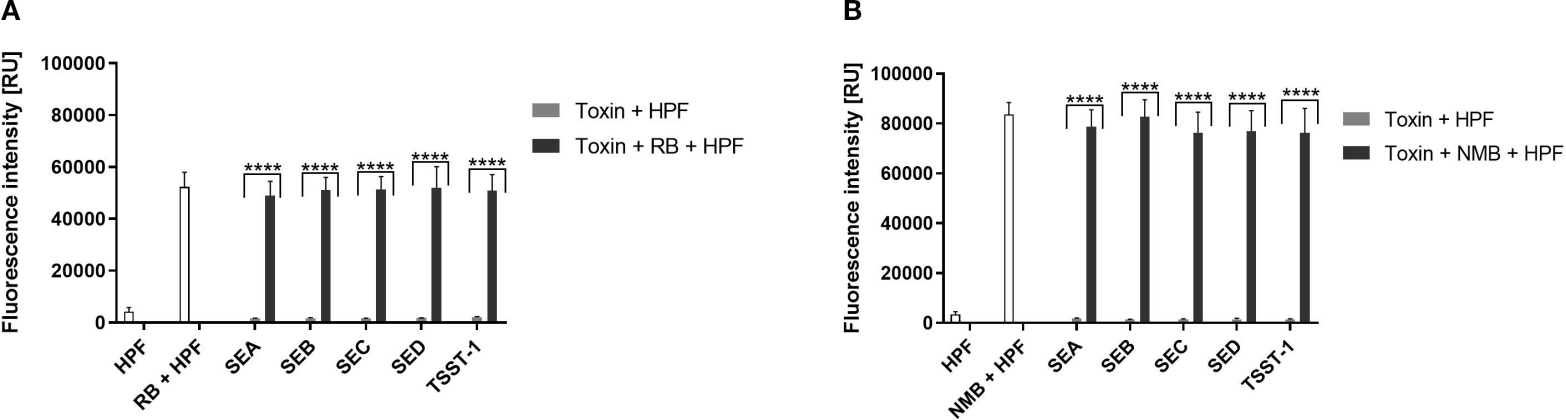
Detection of Reactive Oxygen Species (ROS). **(A)** RB + green light: Cell-free suspensions containing purified staphylococcal enterotoxins (SEA, SEB, SEC SED, TSST-1) and/or rose bengal (RB, 5 µM) were incubated with ROS-sensitive fluorescent probe hydroxyphenyl fluorescein (HPF, 5 µM) to detect hydroxyl radicals (·OH) upon irradiation with green light (λ_max_ = 515 nm, 35 mW/cm², 40 J/cm²). Data are presented as the mean ± standard deviation (SD) from three independent experiments. **(B)** NMB + red light: Cell-free suspensions containing purified enterotoxins and/or new methylene blue (NMB, 5 µM) were incubated with HPF (5 µM) for detection of hydroxyl radicals (·OH) following irradiation with red light (λ_max_ = 632 nm, 20 mW/cm², 32.5 J/cm²). Data are presented as the mean ± SD from three independent experiments. Fluorescence intensity is reported in relative units (RU).

To test ROS generation in our experimental conditions following aPDI treatment, specific probes were employed to detect hydroxyl radicals (•OH). As anticipated, both aPDI treatments; RB + green light and NMB + red light resulted in significant ROS production (**Figures 3A, B**). The fluorescence signal intensity was higher for the NMB + red treatment (~80,000 relative units, RU) compared to RB + green (~50,000 RU) combination. Notably, we did not detect any ROS generation with purified enterotoxins alone (**Figures 3A, B**). Similarly, the results obtained from the dark incubation for both conditions confirmed no ROS production (**Supplementary Figure S3**, **Supplementary Figure S4**).

**Figure 4:**
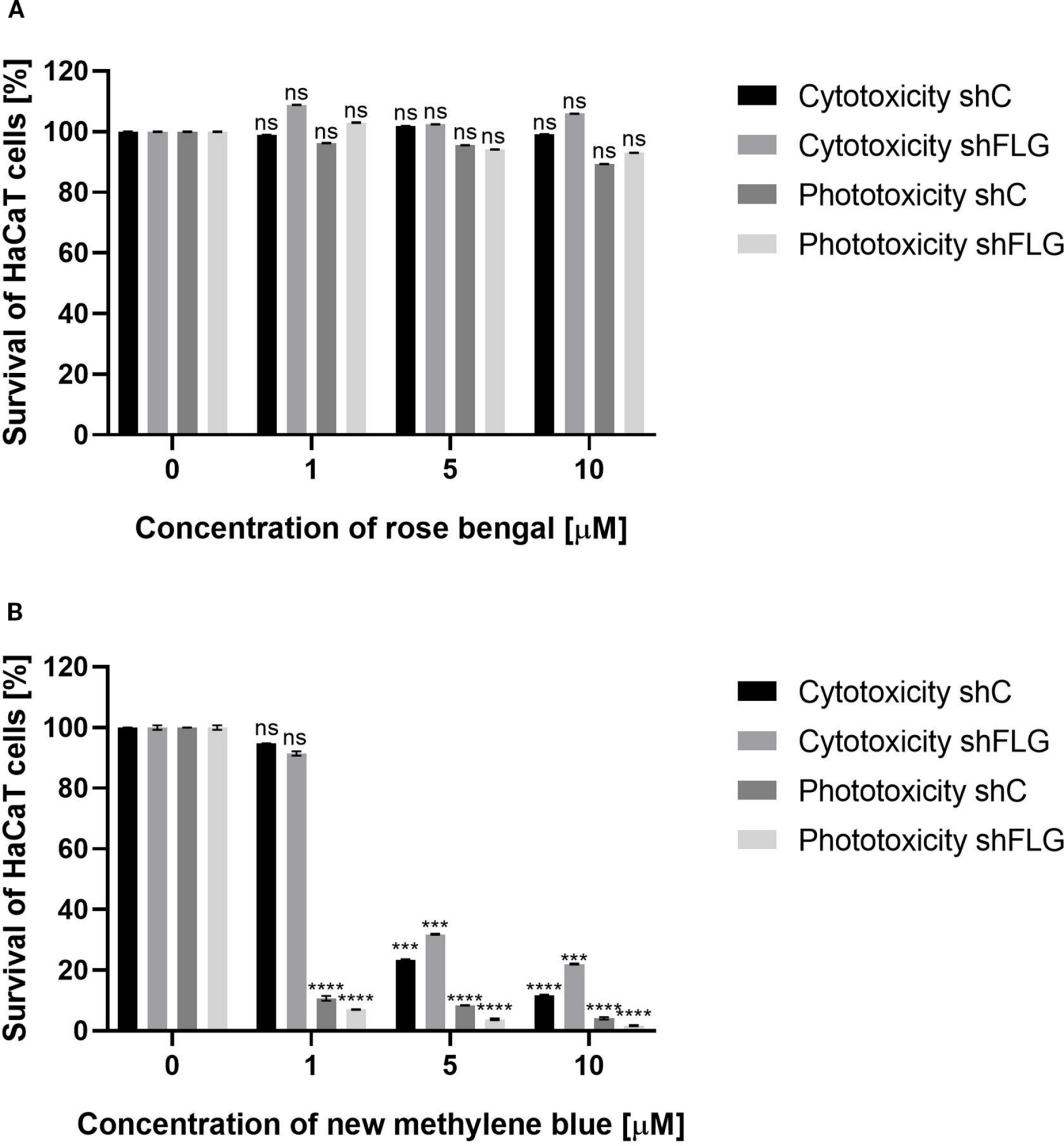
HaCaT cell viability assay. **(A)** Two cell lines – shC HaCaT and shFLG HaCaT keratinocytes were treated with rose bengal (RB) and exposed to green light (λ_max_=515 nm, irradiance 35 mW/cm^2^, light dose 40 J/cm^2^) to test phototoxicity, or kept in the dark to test cytotoxicity. **(B)** The same cell lines were treated with new methylene blue (NMB) and red light (λ_max_=632 nm, irradiance 20 mW/cm^2^, light dose 32.5 J/cm^2^), or kept in the dark. In both experiments, cells (1×10^4^/well) were incubated with increasing photosensitizer concentrations. Untreated cells (0 µM) served as controls. Bars show mean ± SD from three independent experiments. Significant differences *vs*. control are marked (***p < 0.0001; ****p < 0.0001; ns, non significant).

### 3.4 aPDI treatment is safe to human epidermal keratinocytes

Next, we evaluated the impact of aPDI on human epidermal keratinocytes (HaCaT). The analysis included HaCaT cells with a knockdown of the *FLG* gene, which encodes profilaggrin, a crucial protein involved in the maintenance of the epidermal barrier (shFLG HaCaTs). The filaggrin insufficient keratinocytes used in this study served as an *in vitro* model of AD, since filaggrin insufficiency (either on genetic background or acquired, including a vicious loop between filaggrin reduction and *S. aureus* colonization) leads to the impairment in epidermal barrier quality is one of the disease hallmarks (50). As a control cell line transduced with an empty vector was used (shC HaCaT). The primary objective of this experiment was to determine whether the *FLG* knockdown significantly affects the survival of aPDI-treated eukaryotic cells.

MTT assay results demonstrated that the combination of RB with green light (λ_max_ = 515 nm) exhibited no cytotoxic effects on any of the tested cell lines. Cell viability remained high even at the highest RB concentration (10 μM) in the combination with light treatment: 89.3% for shC HaCaT and 93% for shFLG HaCaT cells. Furthermore, treatment with RB alone did not affect cell survival, with the viability values of 99.1% for shC HaCaTs and 106% for shFLG HaCaTs at 10 μM RB (**Figure 4A**).

A very different picture was observed for the combination of NMB with red light (λ_max_ = 632 nm). Exposure to red light with 1 μM NMB resulted in significant cell death, with survival rates of only 10.7% (shC HaCaT) and 7.1% (shFLG HaCaT). In contrast, cells incubated with NMB in the dark showed no cytotoxicity (shC HaCaT – 94.8%, shFLG HaCaT – 91.4%). A strong photo- and cytotoxic effect was observed for both tested HaCaT cell lines at 5 μM NMB (**Figure 4B**). The observed cytotoxic and/or phototoxic effect or lack thereof depends on the PS used in the photodynamic reaction.

### 3.5 aPDI decreases transcript and protein levels of SEA in an *ex vivo* porcine skin model

We next verified the results obtained *in vitro* in an *ex vivo* model, using porcine skin, The primary objectives of this experiment were to assess SEA toxin production at the transcript and protein levels under aPDI. Porcine skin was chosen due to its structural and physiological similarity to human skin and its availability (**Supplementary Figure S5**) (51). A higher RB concentration (35 µM) was used compared to *in vitro* studies due to the limited photosensitizer penetration into the skin and bacterial aggregates hindering binding (49). To evaluate the safety of this approach, we analyzed the cyto- and phototoxicity of RB at higher concentrations and observed some cytotoxic effects (**Supplementary Figure S6**). Nevertheless, higher concentrations were employed because, in *ex vivo* and *in vivo* settings, bacteria are expected to form more resistant biofilm structures that are typically less susceptible to aPDI. The experiment involved colonizing porcine skin with *S. aureus* Xen40 strain, followed by RB application (35 µM) for 30 min, and subsequent green light irradiation (λ_max_ = 530–535 nm, 6.36 J/cm², 10 min). *S. aureus* Xen40 has the *sea* gene and produces active SEA protein.

**Figure 5:**
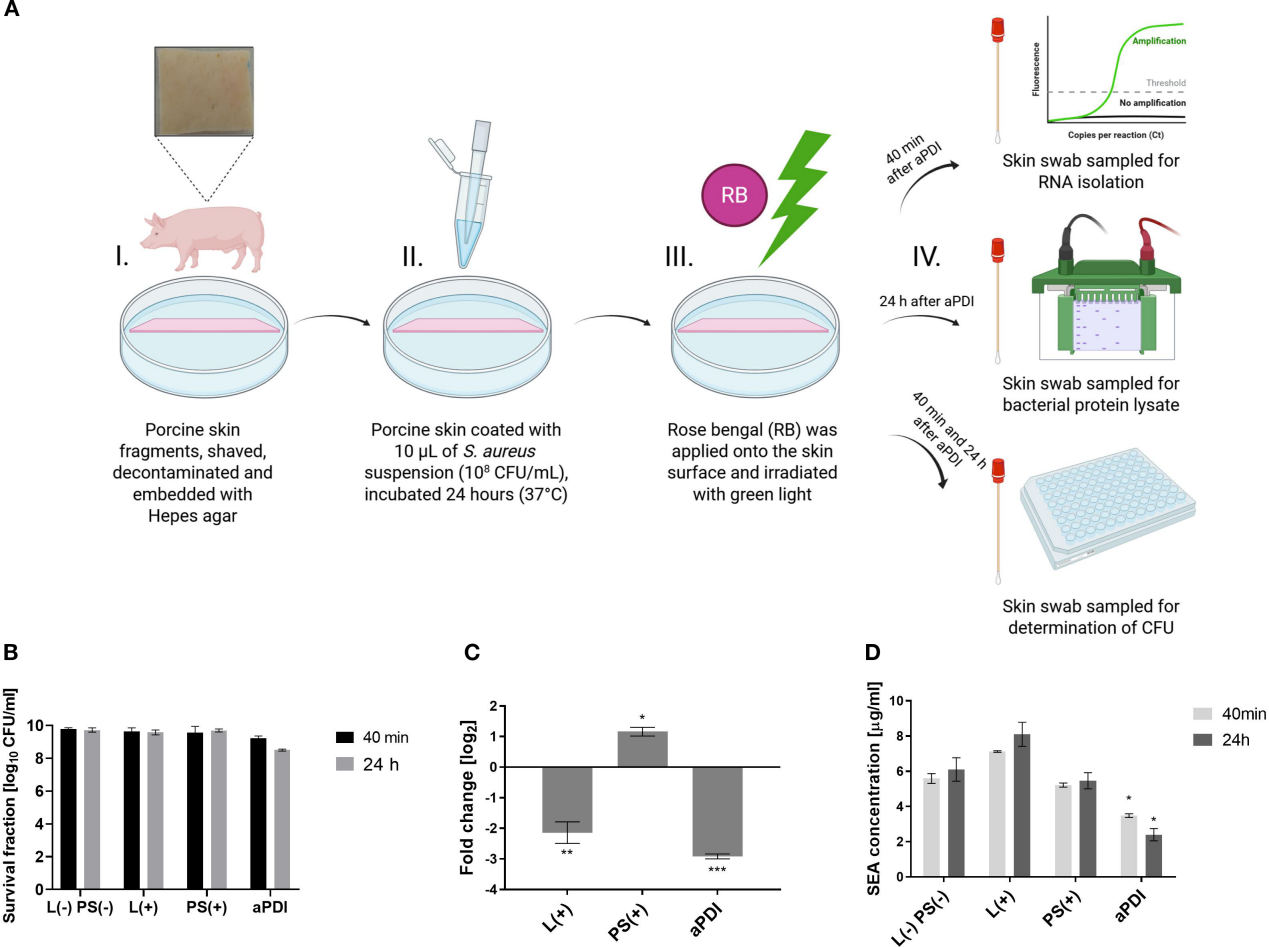
Changes in the SEA toxin levels following aPDI treatment in an *ex vivo* porcine skin colonization model. **(A)** Experimental design - antimicrobial photodynamic inactivation (aPDI) was applied to an *ex vivo* porcine skin model. Illustration created with BioRender. **(B)** Bacterial survival following sublethal aPDI. Values represent the mean log_10_CFU/mL ± SD from three independent biological experiments. **(C)** Relative expression of the *sea* enterotoxin gene after aPDI treatment: Statistical significance compared to untreated cells (0 J/cm^2^, 0 µM photosensitizer [PS]) is indicated by asterisks: *p < 0.05; **p < 0.01; ***p < 0.001. Error bars represent the standard error of the mean (SEM). **(D)** SEA protein concentrations after aPDI: Each bar represents the mean ± SEM from three independent biological replicates. Statistical significance compared to untreated cells is indicated by *p < 0.05. Samples were collected at two time points: 40 min, 24 hours post-aPDI. Treatment groups included: L (–) PS (–), untreated bacterial cells kept in the dark (0 J/cm^2^, 0 µM rose bengal [RB]); L(+),cells exposed to green light only (λ_max_=530–535 nm, 10.6 mW/cm^2^, 6.36 J/cm^2^, 10 min); PS(+),cells treated with RB (35 µM) and kept in the dark; aPDI, cells treated with both RB and green light.

Survival of the *Staphylococcus aureus* Xen40 strain was evaluated at 40 minutes and 24 hours following aPDI. Quantification of bacterial viability revealed a time-dependent reduction in colony-forming units, with decreases of 0.56 log_10_ CFU/mL and 1.22 log_10_ CFU/mL, respectively (**Figure 5B**), indicating a sustained bactericidal effect. The presence of staphylococcal enterotoxin A was confirmed in skin swabs by both PCR and Western blot analysis. Transcriptomic analysis showed a significant downregulation of *sea* gene expression following aPDI treatment, with a reduction of 2.92 log_2_ units (**Figure 5C**). Interestingly, exposure to green light alone also resulted in a notable decrease in *sea* expression (2.14 log_2_ units), suggesting a potential sub-lethal stress response. In contrast, treatment with RB alone led to a significant upregulation of *sea* gene expression, indicating that RB in the absence of light may act as a stressor that induces virulence factor expression. Interestingly, a different effect of gene expression was observed after treatment with RB in an *in vitro* planktonic culture and in an *ex vivo* porcine skin colonization model. In the *in vitro* environment, RB treatment resulted in decreased expression of *sea*, suggesting a possible antimicrobial or stress suppressive effect in a simplified, nutrient-controlled environment. However, in a more complex *ex vivo* model, RB treatment resulted in increased *sea* expression, suggesting that the interaction between bacterial cells and the host-like environment can modulate the expression of virulence genes in different ways.

To further assess the impact of aPDI on enterotoxin production, SEA protein levels were quantified at 40 minutes and 24 hours post-treatment using Western blot analysis. aPDI significantly reduced SEA concentrations compared to untreated controls, with levels decreasing from 5.59 µg/mL to 3.48 µg/mL at 40 minutes, and from 6.10 µg/mL to 2.41 µg/mL at 24 hours (**Figure 5D**). In contrast, treatment with either AD or green light alone did not result in a significant reduction in SEA protein levels, suggesting that the photodynamic activation of RB is essential for the observed effect. Importantly, results from the porcine skin colonization model corroborated the *in vitro* findings, demonstrating consistent reductions in SEA expression at both the transcript and protein levels following aPDI treatment. These data reinforce the potential of aPDI as an effective approach not only for bacterial inactivation but also for mitigating toxin-mediated virulence.

### 3.6 aPDI reduces *S. aureus* survival and SEA toxin level in a mouse model of bacterial skin colonization

In this study, a tape-stripping model was used, this model effectively induces local inflammation by disrupting skin barrier, mimicking atopic skin conditions (52, 53). Previously published *in vivo* studies on aPDI efficacy involved skin wounding and bacterial application to the deeper layers, which does not accurately reflect natural colonization (54). Here, we colonized mechanically damaged mouse skin with bioluminescent *S. aureus* Xen40 to follow its fate after aPDI and identify the presence of staphylococcal enterotoxin A during colonization.

**Figure 6:**
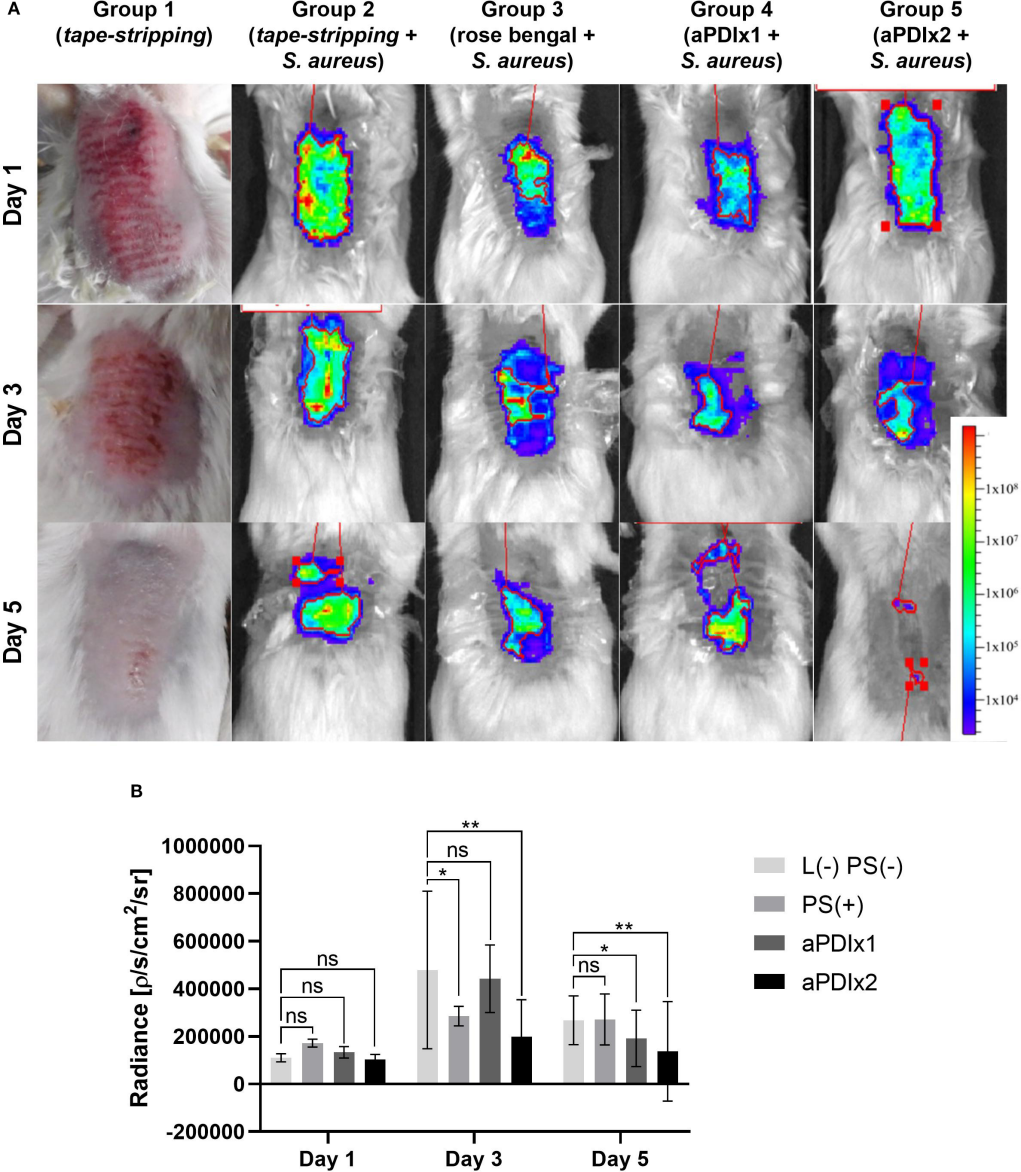
Bioluminescence monitoring of *S. aureus* colonization in a mouse model. **(A)** Representative bioluminescence images of *S. aureus* colonization in mouse skin are shown for one mouse per experimental group. The bioluminescent signal, expressed as photon/second/cm^2^/steradian (p/s/cm^2^/sr), reflects presence of metabolically active bacterial cells. Group 1 illustrates the visual appearance of tape-stripped mouse skin. Group 2 represents tape-stripped mice colonized *S. aureus*. Group 3 includes tape-stripped, *S. aureus*-colonized mice treated with rose bengal (50 µM) applied to the skin. Group 4 consists of *S. aureus*-colonized mice subjected to a single aPDI session (λ_max_=530–535 nm, 10.6 mW/cm^2^, 6.36 J/cm^2^, 50 µM RB). Group 5 represents *S. aureus*-colonized mice receiving two aPDI treatments under the same conditions (λ_max_=530–535 nm, 10.6 mW/cm^2^, 6.36 J/cm^2^, 50 µM RB). **(B)** Quantification of total bioluminescence from the ROI (Region Of Interest) on *S. aureus-*colonized skin. The signal is expressed as a photon/second/cm^2^/steradian (p/s/cm^2^/sr). Each bar represents the mean bioluminescence value from six mice per group, with error bars indicating the standard error of the mean (SEM). Statistical significance compared to the control group (tape-stripped, *S. aureus*-colonized mice) is indicated as *p* < 0.05 (*); *p* < 0.01 (**). L (–) PS (–), tape-stripped, *S. aureus-*colonized; PS(+), tape-stripped, *S. aureus-*colonized mice treated with rose bengal (50 µM); aPDIx1, tape-stripped, *S. aureus-*colonized mice treated with a single aPDI session (50 µM RB, 10.6 mW/cm^2^, 6.36 J/cm^2^); aPDIx2, tape-stripped, *S. aureus-*colonized mice treated with two aPDI sessions (50 µM RB, 10.6 mW/cm^2^, 6.36 J/cm^2^). ns, non significant.

Schematic representation of *in vivo* experimental setup is shown in **Supplementary Figure S7** (Supplementary data). In group 1 (tape-stripping only), skin redness and irritation were observed on day 1, followed by visible healing by day 3 and complete recovery by day 5 (**Figure 6A**). However, in *S. aureus*-colonized skin (group 2), no such healing occurred, indicating that bacterial colonization negatively affects skin condition and delays the healing process (**Supplementary Figure S8**). Both single (group 4) and repeated (group 5) aPDI treatments resulted in a statistically significant reduction in *S. aureus* presence on the skin, as evidenced by decreased bioluminescence signals (**Figure 6A, B**). Although RB alone (without light activation) produced a transient reduction in *S. aureus* signal at day 3, this effect was not sustained over time. Such acute, short-term decreases in bacterial load are occasionally observed in aPDI studies, but durable clearance or inhibition of regrowth typically requires both the photosensitizer and light.

**Figure 7:**
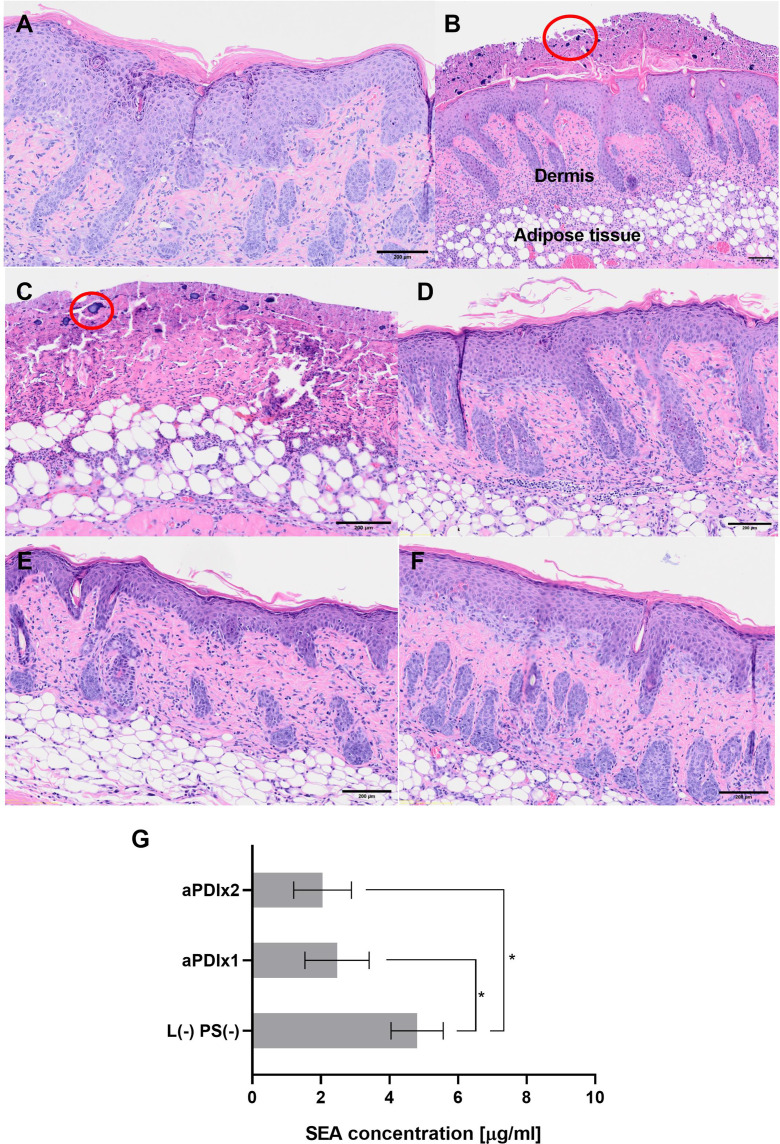
Histological analysis of mouse skin subjected to tape-stripping and *S. aureus* colonization. Histological images of mouse skin samples collected on the 5th day of the experiment. The samples were fixed in formalin, embedded in paraffin, and stained with hematoxylin and eosin (H&E) to assess tissue structure. Bacterial presence was visualized using a crystal violet solution. **(A)** Skin subjected to the tape-stripping procedure (magnification 4×). The red ellipse highlights neutrophil infiltration, indicating an inflammatory response. **(B)** Tape-stripped skin colonized with *S. aureus* (magnification 4×). Clusters of *S. aureus* bacteria are circled in red. **(C)** Tape-stripped, *S. aureus*-colonized skin irradiated with green light (magnification 4×). **(D)** Tape-stripped, *S. aureus*-colonized skin treated with rose bengal (50 μM) applied directly to the skin surface (magnification 4×). **(E)** and **(F)** Tape-stripped, *S. aureus*-colonized skin treated with a single **(E)** or double **(F)** aPDI session (magnification 4×). **(G)** SEA protein concentrations after aPDI. Each bar represents the mean SEA concentration from swab samples collected from six mice, with error bars indicating the standard error of the mean (SEM). Statistical significance is determined in comparison to the control group (tape-stripped, *S. aureus*-colonized mice) with p < 0.05 denoted by (*); aPDIx1 – tape-stripped, *S. aureus*-colonized mice subjected to a single aPDI treatment (50 μM RB, 10.6 mW/cm^2^, 6.36 J/cm^2^); aPDIx2 – tape-stripped, *S. aureus*-colonized mice subjected to a double aPDI treatment (50 μM RB, 10.6 mW/cm^2^, 6.36 J/cm^2^).

Histopathological evaluation of mouse skin revealed distinct tissue responses across experimental groups. In the tape-stripping group (**Figure 7A**), inflammation was evident in the form of exudate with abundant neutrophilic infiltration confined to the epidermis, accompanied by clear evidence of epidermal regeneration, confirming ongoing re-epithelialization. In contrast, mice subjected to tape-stripping followed by colonization with *S. aureus* (**Figure 7B**), exhibited severe pathological alterations. The epidermis was absent, and dense bacterial aggregates were observed on the skin surface. A pronounced neutrophilic infiltrate extended beyond the dermis into subcutaneous adipose tissue and underlying musculature, indicating a robust and deep-seated inflammatory response. Exposure to green light alone (**Figure 7C**) or topical application of RB at a high concentration (50 µM, **Figure 7D**) did not induce additional pathological changes or exacerbate inflammation. No blistering was observed in the group exposed only to light. However, the loss of Tegaderm dressings during the experiment led to increased mechanical irritation and drying of the wounds, which explains the different appearance of the skin in this group. Notably, skin samples from mice treated with a single (**Figure 7E**) or double (**Figure 7F**) aPDI showed no separation of the epidermis from the dermis. In both aPDI-treated groups, neutrophilic infiltration was present in the dermis, with no consistent reduction in neutrophil numbers between single and double treatments. Nevertheless, the inflammatory response remained adequate to control bacterial colonization. These findings suggest that both single and double aPDI treatments are histologically safe and do not aggravate preexisting tissue damage.

Histological images of mouse skin samples collected on the 5th day of the experiment. The samples were fixed in formalin, embedded in paraffin, and stained with hematoxylin and eosin (H&E) to assess tissue structure. Bacterial presence was visualized using a crystal violet solution. **A)** Skin subjected to the tape-stripping procedure (magnification 4×). The red ellipse highlights neutrophil infiltration, indicating an inflammatory response. **B)** Tape-stripped skin colonized with *S. aureus* (magnification 4×). Clusters of *S. aureus* bacteria are circled in red. **C)** Tape-stripped, *S. aureus*-colonized skin irradiated with green light (magnification 4×). **D)** Tape-stripped, *S. aureus*-colonized skin treated with rose bengal (50 µM) applied directly to the skin surface (magnification 4×). **E)** and **F)** Tape-stripped, *S. aureus*-colonized skin treated with a single (E) or double (F) aPDI session (magnification 4×). **G)** SEA protein concentrations after aPDI. Each bar represents the mean SEA concentration from swab samples collected from six mice, with error bars indicating the standard error of the mean (SEM). Statistical significance is determined in comparison to the control group (tape-stripped, *S. aureus*-colonized mice) with *p* < 0.05 denoted by (*); aPDIx1 – tape-stripped, *S. aureus*-colonized mice subjected to a single aPDI treatment (50 µM RB, 10.6 mW/cm², 6.36 J/cm²); aPDIx2 – tape-stripped, *S. aureus*-colonized mice subjected to a double aPDI treatment (50 µM RB, 10.6 mW/cm², 6.36 J/cm²).

The final step of the study involved quantifying the levels of the SEA protein from mouse skin swabs. Three experimental groups were analyzed: (i) a control group consisting of tape-stripped, *S. aureus*-colonized mice, (ii) tape-stripped, *S. aureus*-colonized mice subjected to a single aPDI treatment, and (iii) tape-stripped, *S. aureus*-colonized mice subjected to a double aPDI treatment. In the control group, SEA protein levels were comparable to those observed in both *in vitro* and *ex vivo* studies, confirming consistent toxin production. However, aPDI treatment resulted in a statistically significant reduction in SEA levels, with a progressive trend of decrease following subsequent treatments [L (–) PS (–): 4.8 µg/mL; aPDIx1: 2.47 µg/mL; aPDIx2: 2.05 µg/mL] (**Figure 7G**). These findings demonstrate that aPDI effectively reduces bacterial virulence factors, highlighting its potential as an antimicrobial strategy against *S. aureus*.

## 4 Discussion

*Staphylococcus aureus* is a major contributor to the pathogenesis of atopic dermatitis (AD), where persistent skin colonization and toxin production exacerbate disease severity. Despite the significant impact of *S. aureus* in AD, current treatment options are limited, with few strategies effectively eradicating or controlling its colonization and toxin activity. Our study demonstrates that aPDI effectively reduces *Staphylococcus aureus* colonization in both *in vitro*, *ex vivo* in a porcine skin model, as well as in an *in vivo* murine model, supporting its potential as a non-invasive approach for treating bacterial infections in compromised skin barriers. Our findings align with the previous work by Hamblin’s group, which reported bacterial reduction following RB-mediated aPDI with green light, particularly in the presence of potassium iodide (26). Here, we expanded upon these observations by using multiple models to not only confirm the reduction of the bacterial abundance by aPDI, but also to investigate its modulatory effect on virulence factors, since those play a crucial role in disease severity. A key focus for our study was the impact of aPDI on staphylococcal enterotoxins, i.e., known superantigens that disrupt immune responses and exacerbate skin conditions, such as atopic dermatitis. These findings highlight the aPDI’s potential beyond the antimicrobial strategy but also as a tool for mitigating toxin-associated inflammation in skin infections. While *FLG* mutations represent a strong risk factor for AD, it is important to note that not all mutation carriers develop the disease. The onset of the disease requires the interaction of multiple factors, including genetic background, environmental exposures, and immune regulation, which are required for its manifestation. Our model, therefore, captures only one aspect of this complex interplay, and should be interpreted with these limitations in mind.

Although aPDI has been widely studied for its bactericidal effects, its impact on bacterial virulence factors remains less explored. Previous studies demonstrated that aPDI using methylene blue (MB) and red light (λ_max_ = 660 nm) significantly reduces quorum sensing (QS)-mediated virulence factors in *Serratia marcescens* through downregulation of the *bsmA*, *bsmB*, *flhD*, and *swrR* genes (55). Similarly, aPDI with the use of MB and a diode laser (λ_max_ = 650 nm) suppressed the QS-related genes *lasI*, *lasR*, *rhlI*, and *rhlR* in *Pseudomonas aeruginosa* (56). Comparable gene expression downregulation was observed in *Acinetobacter baumannii* and *S. aureus* when treated with toluidine blue O (TBO) or indocyanine green (ICG) activated by appropriate light sources (57, 58). Additionally, curcumin-mediated aPDI significantly inhibited the expression of key virulence genes (*inlA*, *hlyA*, and *plcA*) in *Listeria monocytogenes* (59). At the protein level, aPDI has been shown to inhibit *S. aureus* V8 protease and α-hemolysin activity in a photosensitizer concentration-dependent manner. Laser-based MB-aPDI (λ_max_ = 665 nm) completely suppressed α-hemolysin activity and reduced sphingomyelinase activity (25). These findings highlight the broad-spectrum potential of aPDI in attenuating bacterial virulence by targeting both gene expression and protein function.

This study provides the first comprehensive assessment of the effect of aPDI strategy on the presence of *S. aureus* enterotoxins in both *in vitro* and *ex vivo* models. Bartolomeu et al. previously demonstrated that enterotoxin-producing *S. aureus* strains were more susceptible to aPDI with Tetra-Py+-Me and white light (380–700 nm) than non-enterotoxigenic strains (26). However, their study was performed exclusively *in vitro* and did not assess the biological activity of enterotoxins post-aPDI.

Our findings reveal that sublethal aPDI with RB and green light, as well as NMB with red light, significantly downregulated the transcript levels of *sea, seb, sec*, and *sed* toxins implicated in *S. aureus*-mediated skin inflammation, particularly in the compromised skin. Interestingly, both the photosensitizers and the light sources independently modulated the expression of certain toxin-encoding genes, aligning with previous reports that blue light (λ_max_ = 462 nm) regulates *S. aureus* motility, iron metabolism, and hemolytic activity (60).

Contrary to previous studies reporting reduced toxin protein levels following aPDI (61, 62), our sublethal treatment conditions did not significantly alter enterotoxin protein levels. This may be attributed to the intrinsic stability of the staphylococcal enterotoxins, which are resistant to heat, desiccation, proteases, and acidic environments. Additionally, factors such as the light dose, photosensitizer concentration, and photobleaching could contribute to the observed resistance. Notably, Tubby et al. reported that aPDI inhibition of *S. aureus* protease activity was highly dependent on the treatment parameters (25). Excessively high photosensitizer concentrations (e.g., 200 µM NMB) may also hinder aPDI effectiveness by absorbing light and reducing ROS generation (37). Prolonged red light exposure (~45 min) can further decrease efficacy due to photobleaching (63). To mitigate this, researchers have proposed administering the photosensitizer in two doses of red light (λ_max_ = 635 ± 15 nm) at 180 J/cm² and 300 J/cm² in *in vivo* studies, achieving a therapeutic effect characterized by the absence of bioluminescence from *Acinetobacter baumannii* (38).

Staphylococcal enterotoxins primarily induce T-cell proliferation and cytokine release (13, 14). Functional assays revealed that while sublethal aPDI slightly impaired toxin activity, lethal aPDI (RB + green light) abolished T-cell proliferation, indicating a significant reduction in toxin-induced immune activation (**Figure 2**). This aligns with the findings by Kömerik et al., who demonstrated that aPDI’s impact on lipopolysaccharide (LPS)-induced cytokine release depended on both photosensitizer concentration and light dose (24). Our results suggest that optimized aPDI parameters could be leveraged as a therapeutic strategy to mitigate *S. aureus* colonization and toxin-mediated inflammation in conditions such as AD.

*Ex vivo* porcine skin models are increasingly used to evaluate aPDI under physiologically relevant conditions. In most previous studies, aPDI was applied immediately after bacterial inoculation, representing short-term protocols that does not allow for bacterial adaptation or colonization comparable to natural *S. aureus* persistence on human skin (49, 64–66). To address this, we developed an *ex vivo* model that allows overnight bacterial colonization, providing a more realistic environment for assessing aPDI’s effects on both transcript and protein levels. Using this model, we demonstrated that RB-mediated aPDI significantly reduced enterotoxin A levels, underscoring its potential for topical applications targeting *S. aureus* virulence (**Figure 5**).

Our study demonstrates that aPDI significantly reduces *S. aureus* colonization and virulence factor production in a murine model that mimics atopic dermatitis. Unlike previous *in vivo* studies that involved deep-tissue infections, our tape-stripping model allowed for the assessment of aPDI’s effects on bacterial colonization in a physiologically relevant setting. Both single and double aPDI treatments resulted in a significant reduction in the bacterial load and/or metabolic activity, as indicated by decreased bioluminescence signals. Histological analysis further confirmed that aPDI did not exacerbate tissue damage; instead, it supported epidermal repair. Importantly, neutrophil numbers were not reduced following aPDI treatment, indicating that the host immune response was preserved (**Figure 7**).

Crucially, aPDI also reduced staphylococcal enterotoxin A levels in a dose-dependent manner, addressing not only the bacterial survival but also toxin-mediated inflammation, an essential factor in skin conditions such as atopic dermatitis (67). This dual effect of aPDI distinguishes it from conventional antimicrobial therapies, which primarily focus on bacterial eradication but may not directly impact the expression or activity of virulence factors. Additionally, aPDI presents a promising alternative to antibiotics, reducing the risk of resistance development. A potential limitation of the system is that aPDI can induce bacterial tolerance under certain conditions. However, this phenomenon appears to be less common and less persistent than the development of antibiotic resistance. Given its non-invasive nature and excellent safety profile, aPDI holds potential for clinical translation in dermatology, particularly for treating *S. aureus*-associated skin diseases. Future research should explore its long-term efficacy, optimal treatment parameters, and its potential synergies with the existing therapies to further enhance its efficacy as an antimicrobial and immunomodulatory intervention.

Our study highlights the ability of aPDI’s to suppress enterotoxin gene expression and, under lethal conditions, significantly reduce the biological activity of the toxin. However, protein-level resistance under sublethal conditions underscores the need for optimized treatment parameters. Future research should explore the impact of different light sources, photosensitizer formulations, and combinatory approaches (e.g., aPDI with antimicrobial peptides) to enhance the efficacy. Additionally, *in vivo* validation in AD models could pave the way for clinical translation of aPDI as a novel strategy against *S. aureus*.

## Data Availability

The original contributions presented in the study are included in the article/**Supplementary Material**. Further inquiries can be directed to the corresponding author.
