# Supplementary material for: Photodynamic inactivation of Staphylococcus aureus with rose bengal reduces superantigen activity
Source: Front Immunol. 2025 Oct 2;16:1655244. doi: 10.3389/fimmu.2025.1655244 (PMC12528143; doi:10.3389/fimmu.2025.1655244)
Supplement: Supplementary file 1 [file DataSheet1.docx]

Photodynamic Inactivation of Staphylococcus aureus with Rose Bengal Reduces Superantigen Activity

Patrycja Ogonowska^1^, Adrian Kobiela^2^, Anna Hulacka^3^, Danuta Gutowska-Owsiak^2^, Joanna Nakonieczna^1^

^1^Laboratory of Photobiology and Molecular Diagnostics, Intercollegiate Faculty of Biotechnology, University of Gdańsk and Medical University of Gdańsk, Poland

^2^Laboratory of Experimental and Translational Immunology, Intercollegiate Faculty of Biotechnology, University of Gdańsk and Medical University of Gdańsk, Poland

^3^Department of Pathomorphology, The University Hospital, Kraków, Poland

**Supplementary Data**

**Determination of sublethal conditions for aPDI-treated *S. aureus***

To define sublethal antimicrobial photodynamic inactivation (aPDI) conditions for downstream analyses, we treated four toxin-producing *S. aureus* strains: 10798/11 (SEA), 140/05 (SEB), 1947/05 (SEC), and 1005 (SED, TSST-1) with rose bengal (RB; 515 nm LED) or new methylene blue (NMB; 632 nm LED). Two experimental regimens were used: one tailored for subsequent gene expression measurements (initial cell concentration ≈10^8^ CFU/mL) and a second tailored for protein-level analyses (≈10^9^ CFU/mL). All strains exhibited high aPDI susceptibility, independent of genotype (**Fig. S1**). Sublethal doses for gene expression analysis, defined as a ~0.5 log₁₀ CFU/mL reductions in bacterial counts, were determined (**Table S1**), with RB/green light, survival decreased by 0.47-0.54 log₁₀ CFU/mL; with NMB/red light,by 0.37-0.5 log₁₀ CFU/mL. Sublethal aPDI doses applied for protein analysis are presented in Table S2. The reduction in bacterial counts following RB/green treatment ranged from 0.64 to 1.32 CFU/mL, whereas for NMB/red it ranged from 0.82 to 1.32 CFU/mL. Light alone (green/red) and dark incubation with a photosensitizer (RB/NMB) did not affect bacterial survival (**Table S1 and Table S2**).

|  | **Gene expression analysis** | **Protein expression analysis** |
| --- | --- | --- |
| **RB + green light** | **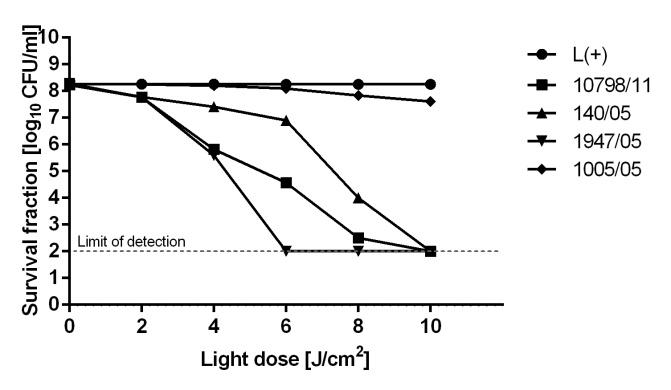** | **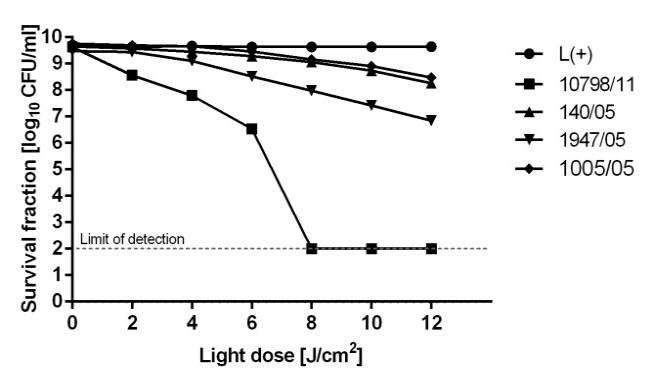** |
| **NMB + red light** | **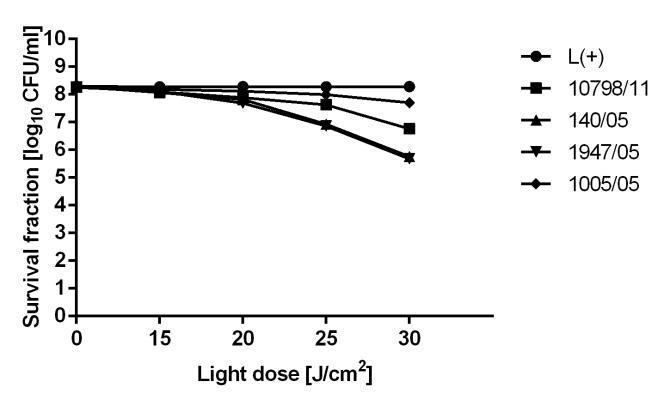** | **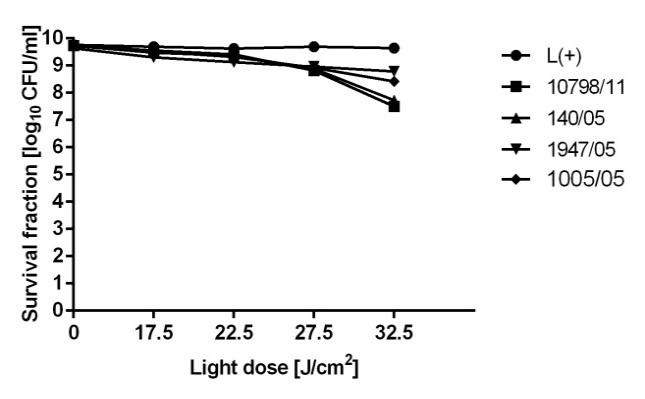** |

**Fig S1. Light-dose dependent antimicrobial photodynamic inactivation of *S. aureus in vitro*.** Light-dose-dependent aPDI of four *S. aureus* strains *in vitro*. Left panel (gene expression regimen; ~10^8^ CFU/mL): λ_max_=515 nm, 35 mW/cm^2^, 0.5 µM RB; λ_max_=632 nm, 20 mW/cm^2^, 5 µM NMB. Right panel (protein level regimen; ~10^9^ CFU/mL): λ_max_=515 nm, 35 mW/cm^2^, 0.5 µM RB; λ_max_=632 nm, 20 mW/cm^2^, 200 µM NMB. Values are means of three independent experiments. Detection limit: 100 CFU/mL. L(+), cells exposed to red/green light only.

**Table S1.** Bacterial survival under sublethal aPDI conditions (for gene expression analysis)

| **NMI collection number** | **Bacterial survival [log_10_ CFU/ml ± SD]^a^** | | | | | | | |
| --- | --- | --- | --- | --- | --- | --- | --- | --- |
|  | **RB + green light** | | | | **NMB + red light** | | | |
|  | **L(-) PS(-)** | **L(+)** | **PS(+)** | **aPDI** | **L(-) PS(-)** | **L(+)** | **PS(+)** | **aPDI** |
| **10798/11** | 8.28±0.02 | 8.28±0.01 | 8.25±0.03 | 7.75±0.16 | 8.29±0.01 | 8.29±0.01 | 8.28±0.02 | 7.92±0.01 |
| **140/05** | 8.25±0.02 | 8.26±0.05 | 8.27±0.02 | 7.78±0.03 | 8.29±0.00 | 8.29±0.01 | 8.29±0.00 | 7.82±0.12 |
| **1947/05** | 8.26±0.00 | 8.28±0.01 | 8.28±0.01 | 7.77±0.01 | 8.28±0.02 | 8.29±0.01 | 8.27±0.01 | 7.82±0.04 |
| **1005/05** | 8.26±0.01 | 8.26±0.05 | 8.25±0.03 | 7.72±0.11 | 8.30±0.00 | 8.27±0.01 | 8.27±0.01 | 7.77±0.04 |

The presented values represent the mean of log_10_ CFU/ml ± SD from three independent biological experiments. L(-) PS(-), untreated bacterial cells kept in the dark (0 J/cm^2^, 0 µM PS); L(+), bacterial cells treated with light only; PS(+), bacterial cells treated with photosensitizer only and kept in the dark; aPDI, treated bacterial cells (light + photosensitizer). NMB, new methylene blue; RB, rose bengal; ^a^the number of bacterial cells transferred into log_10_ CFU/ml.

**Table S2.** Bacterial survival under sublethal aPDI conditions (for analysis at the protein level)

| **NMI collection number** | **Bacterial survival [log_10_ CFU/ml ± SD]^a^** | | | | | | | |
| --- | --- | --- | --- | --- | --- | --- | --- | --- |
|  | **RB + green light** | | | | **NMB + red light** | | | |
|  | **L(-) PS(-)** | **L(+)** | **PS(+)** | **aPDI** | **L(-) PS(-)** | **L(+)** | **PS(+)** | **aPDI** |
| **10798/11** | 9.57±0.04 | 9.57±0.14 | 9.60±0.05 | 8.37±0.02 | 9.53±0.06 | 9.60±0.18 | 9.42±0.07 | 8.35±0.01 |
| **140/05** | 9.67±0.07 | 9.70±0.03 | 9.59±0.09 | 9.03±0.00 | 9.78±0.10 | 9.70±0.12 | 9.73±0.08 | 8.88±0.14 |
| **1947/05** | 9.48±0.06 | 9.37±0.01 | 9.51±0.11 | 7.99±0.03 | 9.72±0.02 | 9.63±0.07 | 9.63±0.10 | 8.79±0.03 |
| **1005/05** | 9.76±0.03 | 9.78±0.15 | 9.76±0.07 | 8.48±0.07 | 9.74±0.07 | 9.76±0.10 | 9.72±0.07 | 8.42±0.02 |

The presented values represent the mean of log_10_ CFU/ml ± SD (standard deviation) from three independent biological replicates. L(-) PS(-), untreated bacterial cells kept in the dark (0 J/cm^2^, 0 µM PS); L(+), bacterial cells treated with light only; PS(+), bacterial cells treated with photosensitizer only and kept in the dark; aPDI, treated bacterial cells (light + photosensitizer). NMB, new methylene blue; RB, rose bengal; ^a^the number of bacterial cells transferred into log_10_ CFU/ml.

**Assessment of toxin protein levels under sublethal aPDI conditions.** The impact of sublethal antimicrobial photodynamic inactivation (aPDI) on toxin protein levels was evaluated using Western blot analysis. The study was conducted on four reference *Staphylococcus aureus* strains, previously validated for toxin production: 10798/11 (SEA), 140/05 (SEB), 1947/05 (SEC), and 1005/05 (SED and TSST-1). Immunodetection was performed, and band intensities corresponding to individual toxin proteins were quantified using Image Lab software (Bio-Rad, USA). Protein concentrations were determined by referencing a standard curve and expressed in µg/ml. The experiment was conducted in three independent biological replicates. The semi-quantitative analysis of immunoblot band intensities revealed that SEB and SEC enterotoxins were produced at the highest levels, reaching maximum concentrations of 35.85 µg/ml and 28 µg/ml, respectively. In contrast, the TSST-1 toxin was detected at low levels (<5 µg/ml), approaching the detection limit of the method. Additionally, a temporal increase in toxin protein concentration was observed over the course of the experiment.

Despite these variations, no significant changes in the level of toxin proteins under sublethal aPDI conditions were observed at all the time points and regardless of the experimental conditions.

|  | | **RB + green light** | | | | | **NMB + red light** | | | | |
| --- | --- | --- | --- | --- | --- | --- | --- | --- | --- | --- | --- |
|  |  | **Standard** | **L(-)**  **PS(-)** | **aPDI** | **L(+)** | **PS(+)** | **Standard** | **L(-)**  **PS(-)** | **aPDI** | **L(+)** | **PS(+)** |
| **SEA** | t0h | 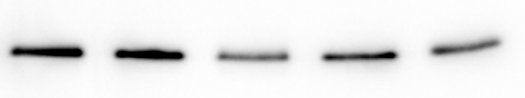 | | | | | 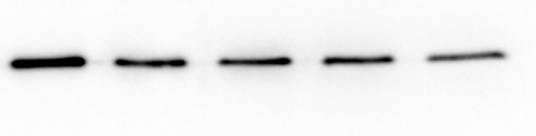 | | | | |
|  | t1h | 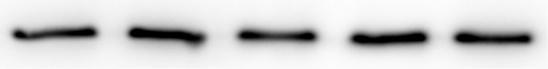 | | | | | 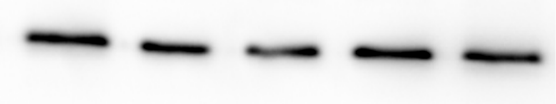 | | | | |
|  | t2h | 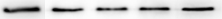 | | | | | 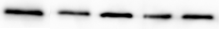 | | | | |
| **SEB** | t0h | 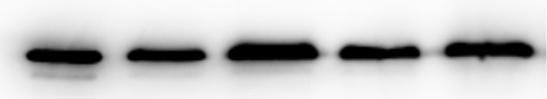 | | | | | 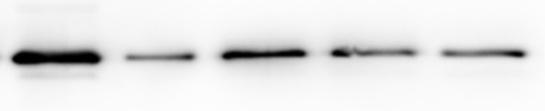 | | | | |
|  | t1h | 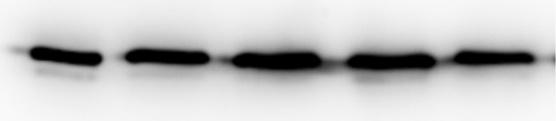 | | | | | 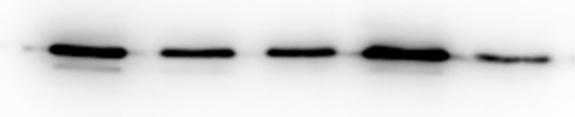 | | | | |
|  | t2h | 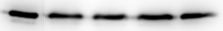 | | | | | 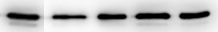 | | | | |
| **SEC** | t0h | 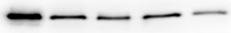 | | | | | 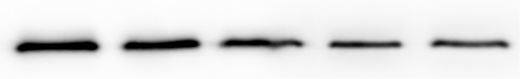 | | | | |
|  | t1h | 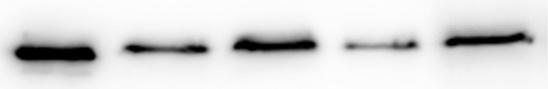 | | | | | 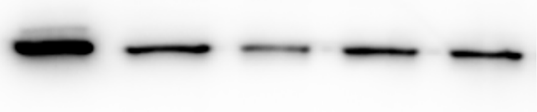 | | | | |
|  | t2h | 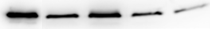 | | | | | 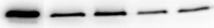 | | | | |
| **SED** | t0h | 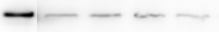 | | | | | 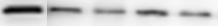 | | | | |
|  | t1h | 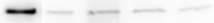 | | | | | 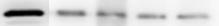 | | | | |
|  | t2h | 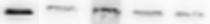 | | | | | 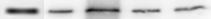 | | | | |
| **TSST-1** | t0h | 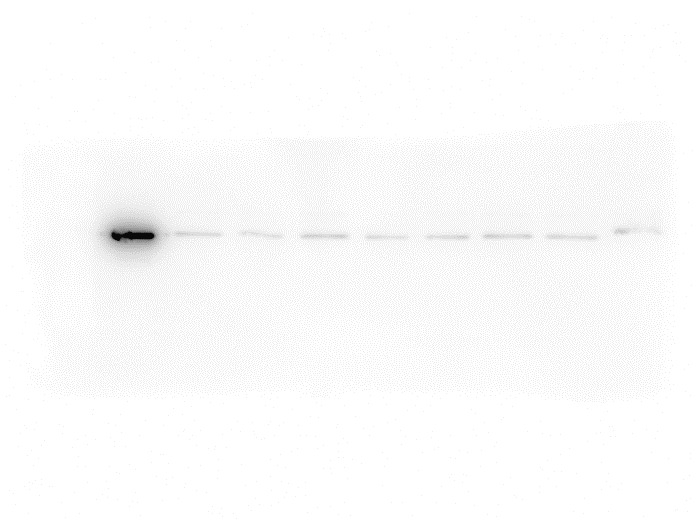 | | | | | 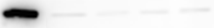 | | | | |
|  | t1h | 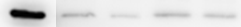 | | | | | 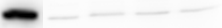 | | | | |
|  | t2h | 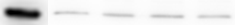 | | | | | 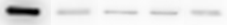 | | | | |

**Fig S2. Image of immunodetection of staphylococcal toxins treated with aPDI under sublethal conditions.** Patterns of the Western Blot bands obtained for one of the three biological replicates are summarized in the table. The results correspond to two treatment conditions: rose bengal (RB) combined with green light (λ_max_=515 nm) (left panel) and new methylene blue (NMB) combined with red light (λ_max_=632 nm) (right panel). Pure toxins at a concentration of 25 µg/ml (5 µl per well) served as standards (Toxin Technology Inc., USA). Bacterial samples were collected at three time points: immediately after the irradiation process (t0h), one (t1h) or two (t2h) hours after the irradiation. L(-) PS(-), untreated bacterial cells; aPDI, cells treated with light and photosensitizer; L(+), cells exposed to light only; PS(+), cells incubated with a photosensitizer and stored in the dark.

**ROS generation during aPDI treatment *in vitro***

Figures S3 and S4 present control experiments demonstrating that reactive oxygen species (ROS) were not generated when proteins were incubated with the photosensitizer alone, in the absence of light activation (referred to as the "dark control").


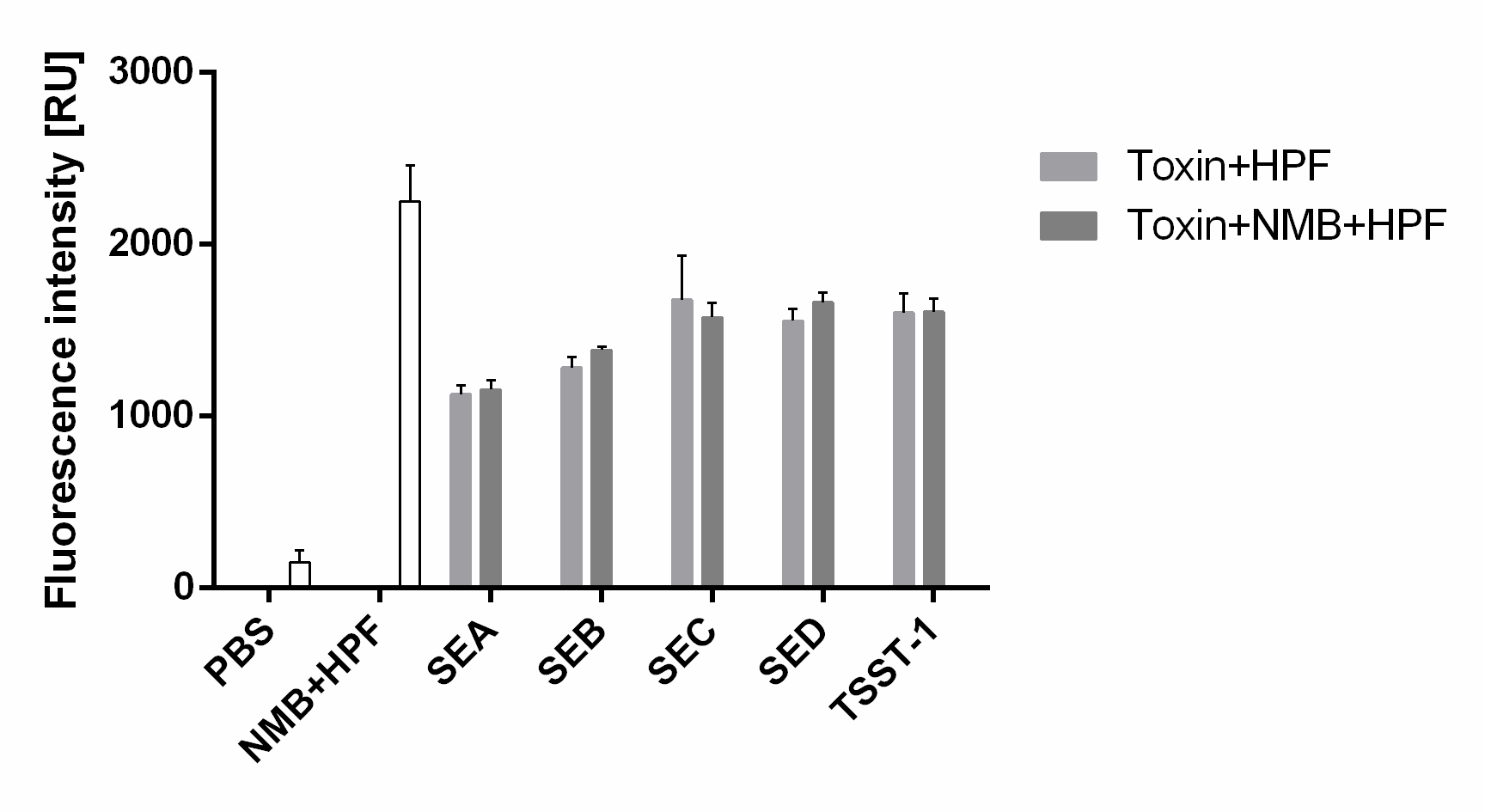


**Fig S3.** ROS detection (NMB + red light). Cell-free suspensions of pure toxins and new methylene blue (NMB) were incubated with ROS-detecting fluorescent probes to detect hydroxyl radicals (·OH) upon storage in the dark. Each result is a mean ± SD of three independent experiments. RU, Relative units; PBS, phosphate buffered saline; NMB, new methylene blue; HPF, hydroxyphenyl fluorescein; SEA, staphylococcal enterotoxin A; SEB, staphylococcal enterotoxin B; SEC, staphylococcal enterotoxin C; SED, staphylococcal enterotoxin D; TSST-1, toxic shock syndrome toxin-1


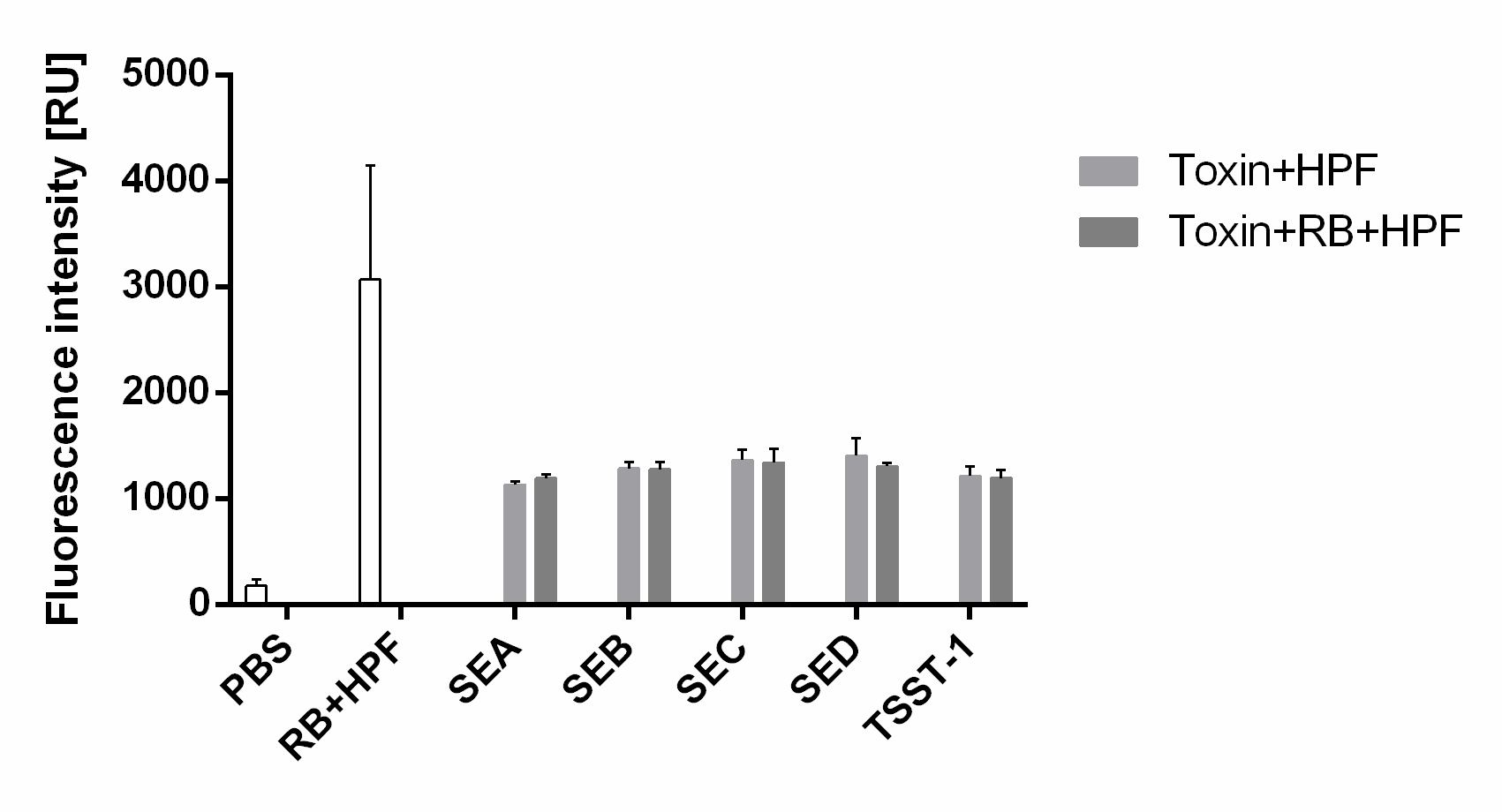


**Fig S4.** ROS detection (RB + green light). Cell-free suspensions of pure toxins and/or rose bengal (RB) were incubated with ROS-detecting fluorescent probes to detect hydroxyl radicals (·OH) upon storage in the dark. Each results is a mean ± SD (standard deviation) of three independent experiments. RU, Relative units; PBS, phosphate buffered saline; RB, rose bengal; HPF, hydroxyphenyl fluorescein; SEA, staphylococcal enterotoxin A; SEB, staphylococcal enterotoxin B; SEC, staphylococcal enterotoxin C; SED, staphylococcal enterotoxin D; TSST-1, toxic shock syndrome toxin-1


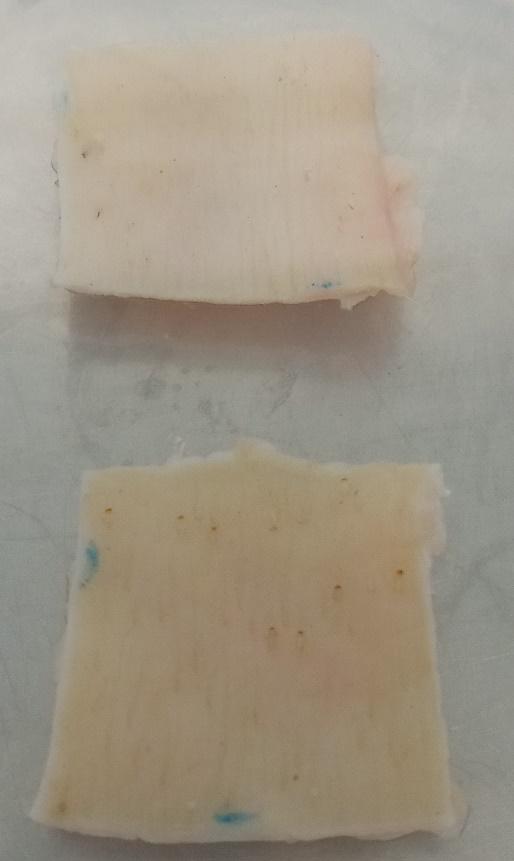

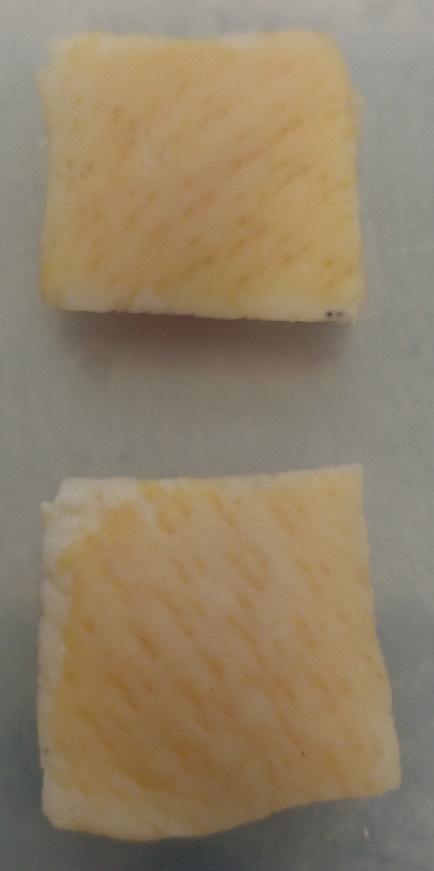


**Fig S5. Porcine skin fragments colonized with *S. aureus*.** On the left – the first day of experiment, porcine skin before the application of *S. aureus*. On the right – porcine skin after 24 hours of incubation, discoloration of the skin surface was observed and skin fragments were colonized with *S. aureus*


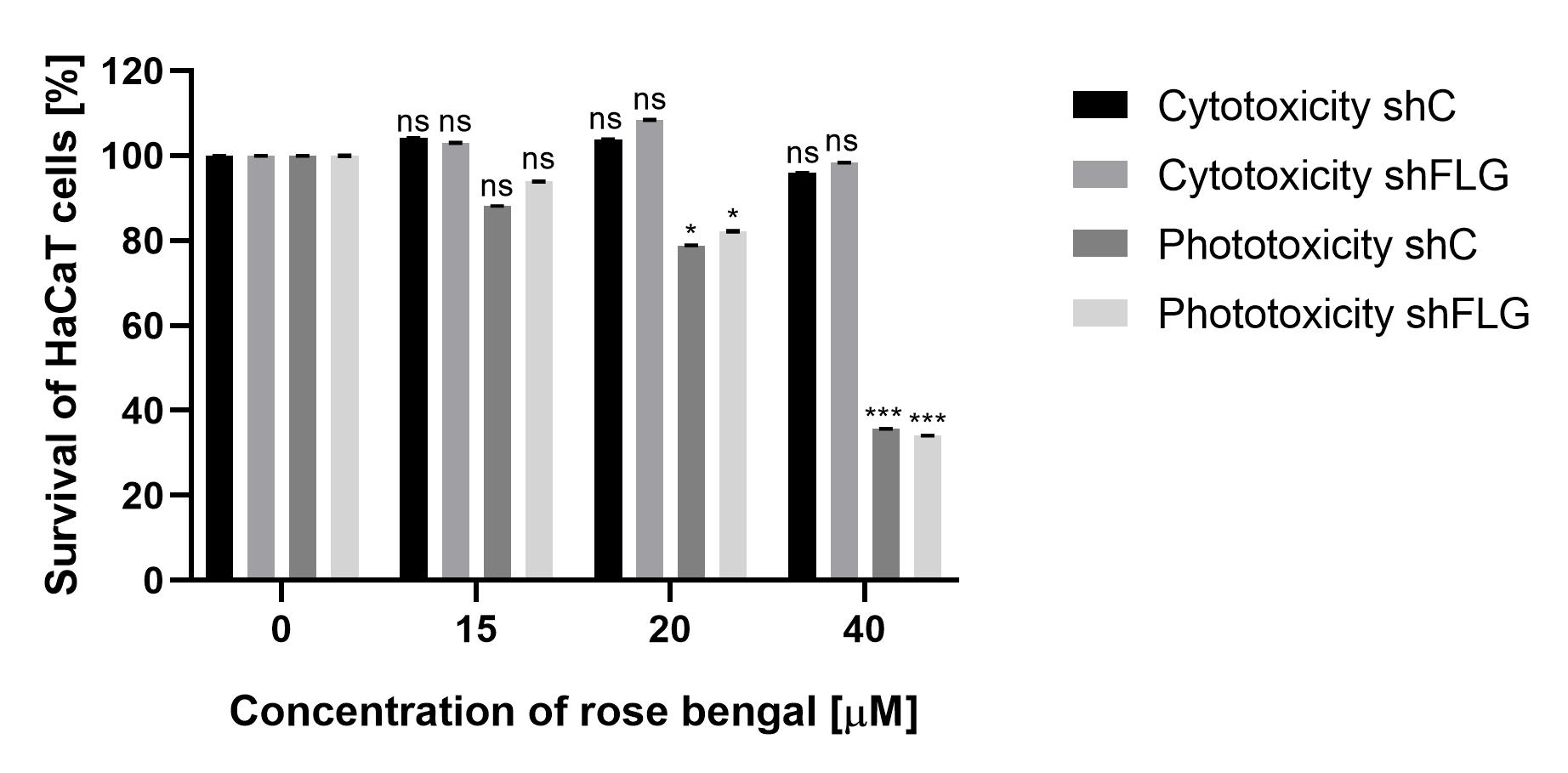


**Fig. S6**. Viability of HaCaT keratinocytes following treatment with rose bengal (RB) and illumination. Two HaCaT-derived cell lines, shC HaCaT and shFLG HaCaT, were incubated with increasing concentrations of RB and either exposed to green light (λmax = 530–535 nm, light dose: 6.36 J/cm²) to assess phototoxicity, or maintained in the dark to assess cytotoxicity. Cells were seeded at 1 × 10⁴ per well, and untreated cells (0 µM RB) served as controls. Data represent mean ± SD from three independent experiments. Statistical significance relative to control is indicated (*p < 0.05; ***p < 0.0001).


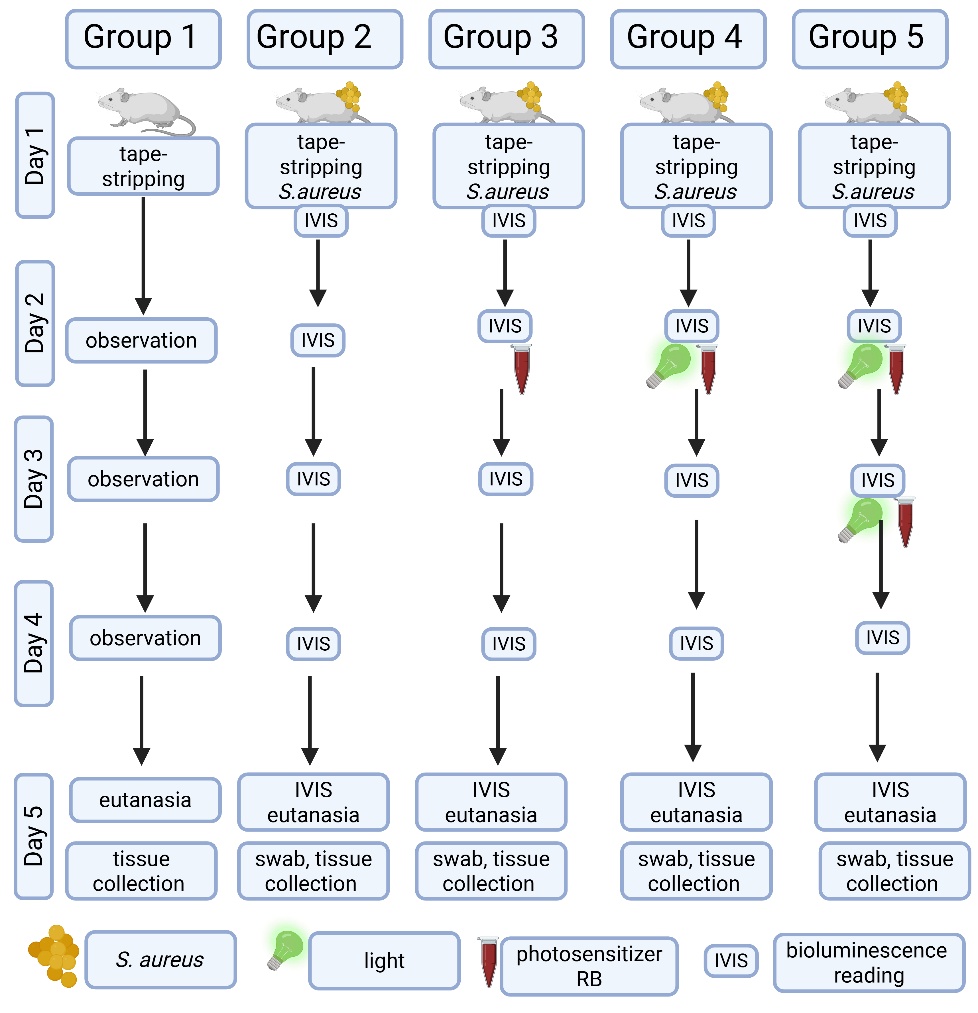


**Fig. S7** Schematic representation of *in vivo* experimental setup.


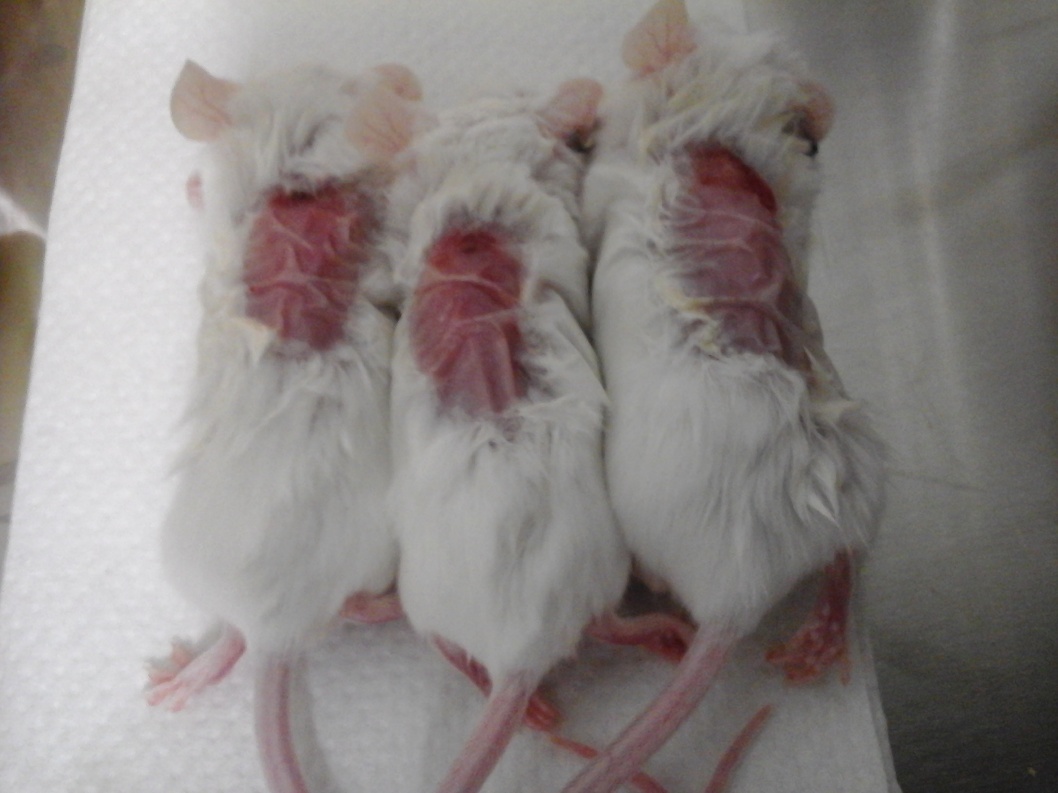

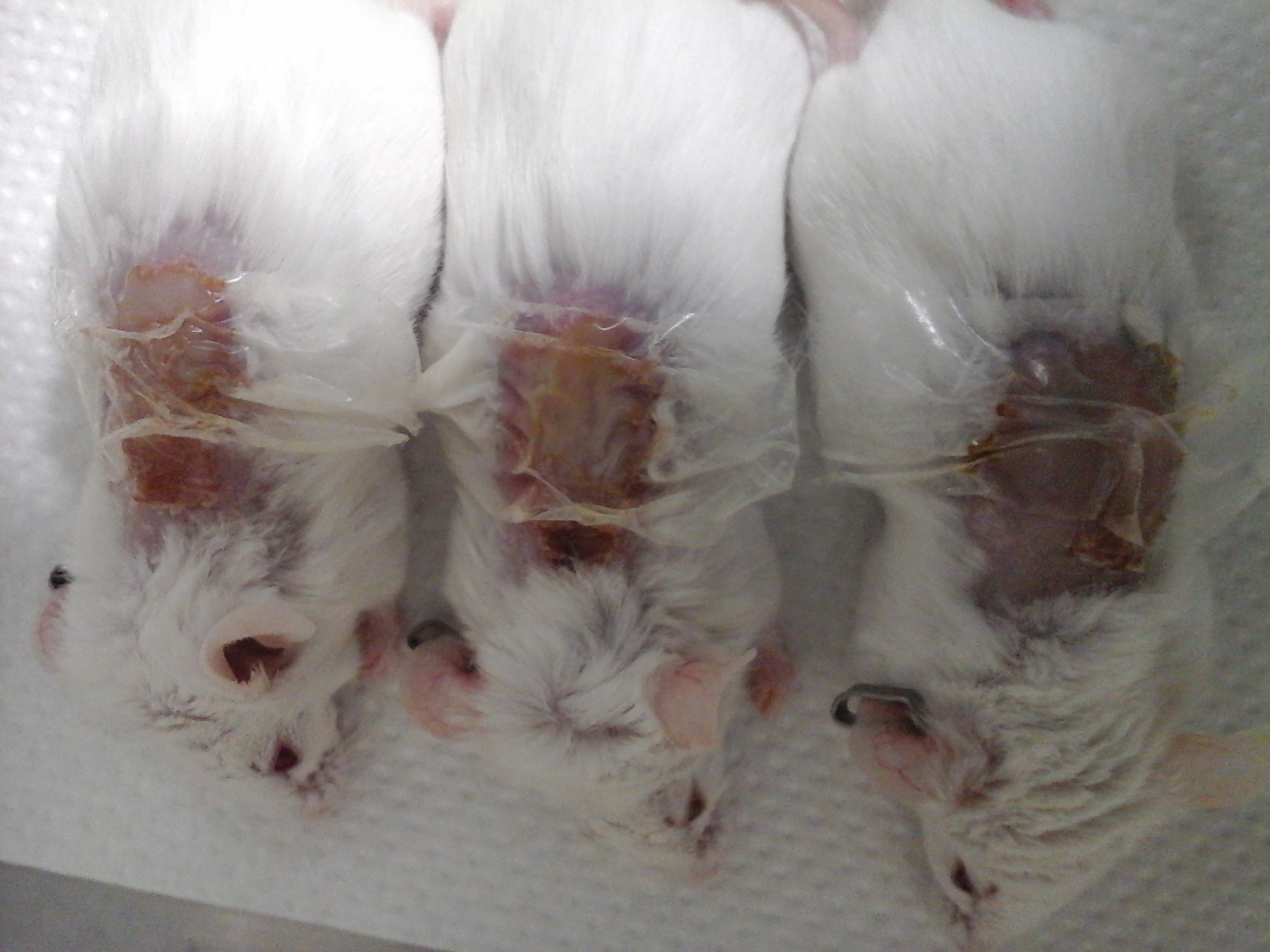

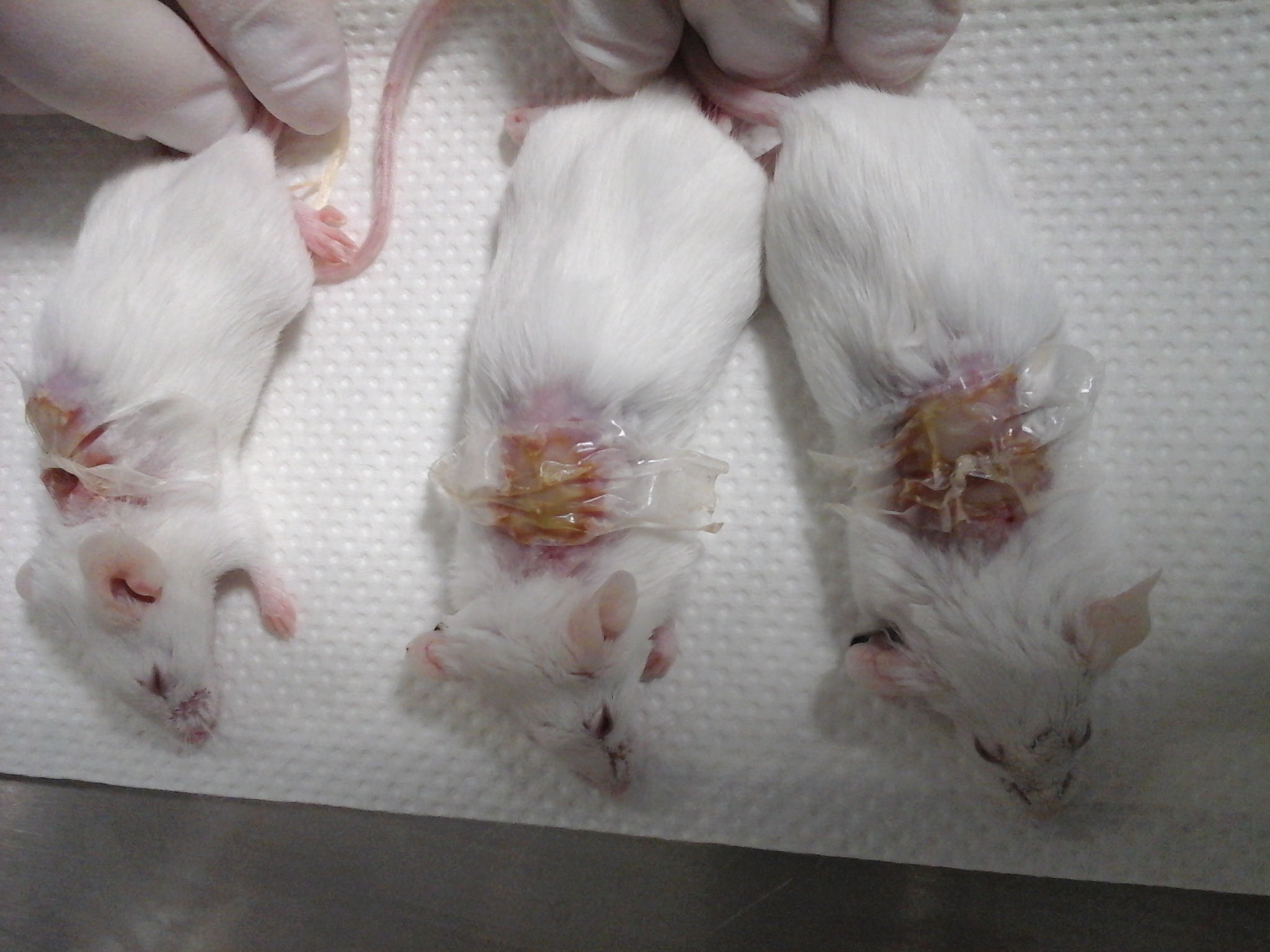


Day 1 Day 3 Day 5

**Fig. S8.** The photos show three representative mice that underwent tape stripping and were infected with *S. aureus*, followed for 5 days to monitor infection development. The wrinkles on the skin surface and slight light reflections are due to the presence of Tegaderm patches.

**Table S3.** qPCR efficiency and slope values of the standard curves of each target gene and reference gene.

| **NMI collection number** | **Gene** | **Efficiency** | **Efficiency (%)** | **Slope** |
| --- | --- | --- | --- | --- |
| **10798/11** | *fabD* | 2.099 | 109.9 | -3.105 |
|  | *ftsZ* | 2.020 | 102 | -3.269 |
|  | *gmk* | 1.925 | 92.5 | -3.515 |
|  | *proC* | 1.930 | 93 | -3.503 |
|  | *sea* | 2.021 | 102.1 | -3.273 |
| **140/05** | *fabD* | 2.109 | 110.9 | -3.086 |
|  | *ftsZ* | 1.961 | 96 | -3.418 |
|  | *gmk* | 2.077 | 107.7 | -3.150 |
|  | *proC* | 1.931 | 93.1 | -3.500 |
|  | *seb* | 2.084 | 108.4 | -3.136 |
| **1947/05** | *fabD* | 2.036 | 103.6 | -3.238 |
|  | *ftsZ* | 2.099 | 110 | -3.105 |
|  | *gmk* | 2.044 | 104.4 | -3.221 |
|  | *proC* | 2.010 | 101 | -3.297 |
|  | *sec* | 2.024 | 102.4 | -3.265 |
| **1005/05** | *fabD* | 2.077 | 107.7 | -3.151 |
|  | *ftsZ* | 1.927 | 92.7 | -3.511 |
|  | *gmk* | 2.021 | 102.1 | -3.273 |
|  | *proC* | 2.083 | 108.3 | -3.137 |
|  | *sed* | 2.065 | 106.5 | -3.176 |
|  | *tst* | 2.065 | 106.5 | -3.175 |
